# Curcumin Inhibits Protease Activated Receptor 2-Induced ERK Phosphorylation Calcium Mobilization and Anti-Apoptotic Signaling in Inflammation-Driven Colorectal Cancer Cells

**DOI:** 10.3390/cells14181451

**Published:** 2025-09-16

**Authors:** Rajashree Patnaik, Riah Varghese, Ahad Al-Kabani, Shirin Jannati, Yajnavalka Banerjee

**Affiliations:** 1 College of Medicine and Health Sciences, Mohammed Bin Rashid University of Medicine and Health Sciences (MBRU), Dubai Health, Dubai 505055, United Arab Emiratesriah.varghese@dubaihealth.ae (R.V.); ahad.alkabani@students.mbru.ac.ae (A.A.-K.); shirin.jannati@students.mbru.ac.ae (S.J.); 2 Department of Basic Medical Sciences, Mohammed Bin Rashid University of Medicine and Health Sciences (MBRU), Dubai Health, Dubai 505055, United Arab Emirates

**Keywords:** curcumin, PAR-2 signaling, colorectal cancer, ERK phosphorylation, calcium mobilization, apoptosis, *Caspase-8*, TNF-α, molecular docking, GPCR modulation

## Abstract

Background: Chronic inflammation drives colorectal cancer (CRC) progression, with PAR-2, a G-protein coupled receptor, linking extracellular inflammatory signals to tumor-promoting pathways via ERK1/2 phosphorylation, calcium mobilization, TNF-α upregulation, and apoptosis suppression. While curcumin has notable anti-inflammatory and anti-cancer properties, its effects on PAR-2 signaling in inflammation-driven CRC remain underexplored. Objective: This study investigates how curcumin modulates PAR-2 expression and downstream oncogenic signaling in inflammation-driven CRC cells and explores its potential direct interaction with PAR-2 at the structural level. Methods: HT 29 and Caco-2 CRC cell lines were exposed to lipopolysaccharide (LPS) to induce an inflammatory phenotype, followed by treatment with curcumin at 50 µM and 100 µM. PAR-2 and PAR-1 expression, along with downstream markers including ERK1/2, p-ERK, TNF-α, *caspase-8*, cleaved *caspase-8*, *caspase-3*, *Bcl 2*, and *Bax*, were analyzed by Western blot and quantitative PCR. Calcium mobilization was assessed using Fluo-4 dye-based fluorescence imaging. Apoptosis was quantified using MTT viability assays, AO/EtBr dual staining, and Annexin V/PI flow cytometry. In parallel, AlphaFold-predicted structural models of PAR-2 were used to perform molecular docking with curcumin using CB-Dock2, to identify potential binding pockets and assess binding energetics. Results: Curcumin selectively downregulated PAR-2—but not PAR-1—at both transcript and protein levels in a dose-dependent manner. This downregulation was accompanied by suppression of ERK phosphorylation and calcium signaling, inhibition of TNF-α secretion, and reversal of the anti-apoptotic signaling axis (*Bcl 2* downregulation and *Bax* and *caspase-3*/*-8* upregulation). Functional assays confirmed enhanced apoptosis in curcumin-treated cells. Computational docking revealed a high-affinity binding interaction between curcumin and the transmembrane domain of PAR-2, supporting the hypothesis of direct G-Protein-Coupled Receptor (GPCR) modulation. Conclusions: Our findings reveal that curcumin targets the PAR-2/ERK/TNF-α axis and reactivates apoptotic pathways in inflammation-driven CRC, establishing it as a potent, mechanistically validated candidate for therapeutic repurposing in CRC.

## 1. Introduction

Colorectal cancer (CRC) represents a significant global health burden, ranking among the most diagnosed malignancies and a leading cause of cancer-related mortality worldwide [1]. The pathogenesis of CRC is complex, involving a multi-step process driven by the accumulation of genetic and epigenetic alterations [2,3]. Traditionally, two main molecular pathways have been described: the conventional adenoma-carcinoma sequence [4], characterized by mutations in genes such as *APC*, *KRAS*, and *TP53*, and the serrated pathway [5], often involving *BRAF* mutations and CpG island methylator phenotype (CIMP), including *MLH1* methylation. However, accumulating evidence strongly implicates chronic inflammation as a pivotal factor in the initiation, promotion, and progression of CRC, acting as a critical driver alongside these genetic abnormalities [6].

The link between inflammation and CRC is most clearly exemplified by colitis-associated cancer (CAC), which develops in patients with chronic inflammatory bowel diseases (IBD), such as ulcerative colitis (UC) and Crohn’s disease [7,8,9]. Patients with IBD have a higher risk of CRC, with CAC often showing earlier onset, distinct tumor sites, and unique molecular features, though some overlap with sporadic CRC exists [10]. Beyond IBD, chronic inflammation stemming from other sources, including certain infections or conditions associated with systemic inflammation like obesity and diabetes [11], also contributes to CRC risk, suggesting inflammation serves as a common mechanistic thread linking diverse etiological factors to colorectal carcinogenesis.

The mechanisms by which chronic inflammation fuels CRC are multifaceted [12]. The persistent inflammatory microenvironment elevates reactive oxygen and nitrogen species, causing oxidative stress, DNA damage, and genomic instability (MSI and CIN), thereby promoting tumor initiation [3]. Furthermore, epigenetic modifications, such as the hypermethylation of promoter regions for tumor suppressor and DNA mismatch repair genes, represent another layer of inflammation-induced genomic dysregulation [13]. The inflammatory milieu is rich in cytokines (e.g., TNF-α, IL-6, IL-1β) and growth factors, mainly secreted by infiltrating immune cells such as macrophages and lymphocytes within the TME [14]. These mediators activate critical intracellular signaling pathways in epithelial cells, notably the nuclear factor-kappa B (NF-κB) and signal transducer and activator of transcription 3 (STAT3) pathways. Constitutive NF-κB and STAT3 activation, often driven by IL-6 within the TME, induces genes for proliferation (e.g., Cyclin D1), survival (e.g., *Bcl 2*, survivin), angiogenesis (e.g., VEGF), and inflammation, creating feed-forward loops that sustain tumor growth [3]. The crosstalk between NF-κB and STAT3 orchestrates interactions between malignant cells and the inflammatory TME, a complex ecosystem of cancer cells, immune cells, fibroblasts, endothelial cells, signaling molecules, and extracellular matrix that actively drives carcinogenesis. Tumors sustain chronic inflammation by releasing pro-inflammatory factors and recruiting immunosuppressive cells, reshaping the TME to promote growth, invasion, and metastasis. This bidirectional interplay highlights the TME as a key determinant of CRC progression, suggesting that targeting this environment offers significant therapeutic potential [15].

Natural products represent a valuable source for identifying novel agents for cancer prevention and treatment [16]. Curcumin, a hydrophobic polyphenol derived from the rhizome of *Curcuma longa* (turmeric), has been used for centuries in traditional Indian and Chinese medicine and is recognized for its potent antioxidant, anti-inflammatory, and anti-cancer properties [17]. Extensive preclinical research supports the potential of curcumin as a chemopreventive and therapeutic agent against various malignancies, including CRC [18]. Its hydrophobic nature allows it to readily diffuse across cellular membranes, reaching intracellular compartments like the endoplasmic reticulum, mitochondria, and nucleus to exert its pleiotropic effects [19].

In CRC, curcumin modulates multiple molecular targets and signaling pathways involved in inflammation and carcinogenesis. Its anti-inflammatory effects include inhibiting transcription factors such as NF-κB—partly by blocking IκB kinase (IKK) activation and NF-κB nuclear translocation—and activator protein 1 (AP-1) [20,21]. Curcumin also downregulates the expression and activity of pro-inflammatory enzymes such as cyclooxygenase-2 (COX-2), often selectively over COX-1, and reduces the production of inflammatory cytokines including TNF-α and IL-6 [22,23,24]. Beyond its direct anti-inflammatory actions, curcumin interferes with multiple oncogenic signaling cascades crucial for CRC development and progression [25]. Studies have shown its ability to inhibit the JAK/STAT3 pathway [26,27], various arms of the mitogen-activated protein kinase (MAPK) pathways including extracellular signal-regulated kinase (ERK) [28,29], c-Jun N-terminal kinase (JNK) [30], and p38 MAPK [31], the phosphoinositide 3-kinase (PI3K)/protein kinase B (Akt) pathway [32], and the Wnt/β-catenin signaling pathway [33]. By targeting these central nodes, curcumin can effectively suppress proliferation, survival signals, invasion, and metastasis.

Furthermore, curcumin is a potent inducer of apoptosis in CRC cells, acting through both intrinsic and extrinsic pathways [34]. Mechanistically, this involves the activation of key executioner caspases (e.g., *caspase-3*, *-7*) [35,36], and initiator caspases (e.g., *caspase-8*, *-9*) [37,38], leading to the cleavage of substrates like poly (ADP-ribose) polymerase (PARP) [39]. Curcumin also modulates the balance of *Bcl 2* family proteins, typically downregulating anti-apoptotic members (e.g., *Bcl 2*, *Bcl-xL*) while potentially upregulating pro-apoptotic members (e.g., *Bax*) [40,41]. Additional mechanisms contributing to its pro-apoptotic effects may include the generation of reactive oxygen species (ROS) and induction of endoplasmic reticulum (ER) stress [42]. Curcumin also induces cell cycle arrest (G1/S or G2/M) [43], inhibits proliferation, modulates gut microbiota [44], targeting cancer stem cells (CSCs) [45], influencing epigenetic modifications [46], and inhibiting angiogenesis [47].

Despite extensive data on curcumin’s intracellular targets, its impact on cell surface receptors like GPCRs, key transducers of diverse extracellular signals into intracellular responses, remains poorly understood [48]. GPCRs are critical regulators of numerous physiological processes, including inflammation and metabolism, and their dysregulation is implicated in various diseases, making them major drug targets [49]. Given curcumin’s ability to permeate cell membranes and its documented impact on signaling pathways often downstream of GPCR activation (e.g., MAPK pathways, calcium signaling), exploring its potential interactions with specific GPCRs relevant to CRC pathogenesis is warranted. However, direct evidence specifically linking curcumin to the modulation of GPCR family members is currently limited. This represents a notable gap in understanding, particularly considering the established importance of GPCRs in cancer biology [50,51]. Curcumin’s inhibition of NF-κB and ERK, key effectors of pro-tumorigenic GPCRs, suggests it may antagonize GPCR pathways in CRC. Our recent work in a pro-inflammatory chondrocyte model revealed that curcumin markedly downregulates PAR-2 expression and associated cytokines (TNF-α, IL-6, IL-8), with siRNA studies confirming PAR-2 specificity [52]. These findings provide the first evidence of curcumin’s direct modulation of a GPCR, highlighting its potential as a PAR-2 antagonist in inflammation and cancer.

PAR-2, encoded by the *F2RL1* gene, has emerged as a key player in CRC [53,54]. PAR-2, a unique GPCR, is activated by proteolytic cleavage of its extracellular N-terminus by serine proteases (e.g., trypsin, mast cell tryptase, factors VIIa and Xa), exposing a tethered ligand (SLIGKV in humans) that triggers conformational changes and downstream G-protein signaling [55]. Compelling evidence indicates that PAR-2 expression is significantly upregulated in human CRC tissues compared to adjacent normal colonic mucosa [56]. Analysis of large cancer datasets reveals that *F2RL1* expression is among the highest in colon adenocarcinoma compared to numerous other cancer types [57], and significantly higher than other PAR family members (*F2R*/PAR1, *F2RL2*/PAR3, *F2RL3*/PAR4) within CRC tissues [58]. This elevated expression often correlates with more aggressive tumor phenotypes, advanced disease stage, and consequently, poorer patient prognosis, positioning PAR-2 as a potential prognostic marker.

Functionally, PAR-2 acts as a critical mediator linking the inflammatory TME to CRC progression. Proteases released by immune cells, stromal cells, or even gut microbiota within the inflamed colon can activate PAR-2 on cancer cells and surrounding epithelial cells [59]. PAR-2 activation contributes directly to the pro-inflammatory milieu by stimulating the release of cytokines like TNF-α [53], and can disrupt intestinal epithelial barrier integrity, potentially through mechanisms involving receptor endocytosis and sustained endosomal signaling, further perpetuating inflammation and associated pain [60]. Crucially, PAR-2 signaling engages key intracellular pathways that drive tumorigenesis. Activation consistently leads to the phosphorylation and activation of the MAPK/ERK pathway (ERK1/2) [61], a central regulator of cell proliferation and survival. PAR-2 signaling also triggers mobilization of intracellular calcium ([Ca^2+^]i) [53], a versatile second messenger implicated in regulating ERK activation, proliferation, migration, and apoptosis. Aberrant calcium signaling itself is increasingly recognized as a contributor to cancer progression. Furthermore, PAR-2 signaling intersects with other critical oncogenic pathways, including NF-κB [62], and the Wnt/β-catenin pathway, potentially via interactions involving LRP6 and Axin that lead to β-catenin stabilization and transcriptional activation of target genes involved in cell proliferation and cancer stem cell maintenance [53].

PAR-2 activation drives key cancer hallmarks, including proliferation, migration, invasion, EMT, and angiogenesis via ERK-or p38 MAPK-mediated upregulation of VEGF and COX-2. Notably, PAR-2 also promotes chemoresistance, a major challenge in CRC treatment [53]. PAR-2 activation in CRC cells reduces doxorubicin-induced apoptosis by suppressing ROS generation and *caspase-8/-3* activation, while upregulating anti-apoptotic proteins Mcl-1 and *Bcl-xL* via ERK1/2. Genetic deletion or pharmacological blockade of PAR-2 restores doxorubicin sensitivity. These findings indicate that PAR-2 acts as a key signaling hub in inflammatory CRC, linking the protease-rich TME to pathways driving proliferation, survival, invasion, angiogenesis, and therapy resistance. Its inhibition reverses chemoresistance, and its preferential upregulation over PAR-1 makes PAR-2 an attractive therapeutic target [57]. and by preclinical studies showing that selective PAR-2 modulation without affecting PAR-1 is achievable [53,54], potentially avoiding off-target effects associated with broader PAR inhibition (e.g., on coagulation mediated by PAR-1) [63]. However, no PAR-2 inhibitors are clinically available for CRC, underscoring an urgent need [54]. This study addresses this gap by investigating whether curcumin directly suppresses PAR-2-driven inflammatory signaling, thereby revealing a novel therapeutic mechanism in inflammation-linked malignancies. Using human CRC cell lines HT 29 and Caco-2, representing distinct CRC phenotypes, we systematically examined curcumin’s effects on PAR-2 expression and downstream pathways under an LPS-induced inflammatory environment [64,65].

Our investigation revealed several key findings. Treatment with curcumin significantly attenuated the LPS-induced upregulation of PAR-2 expression at both the mRNA and protein levels in both HT 29 and Caco-2 cells. Importantly, this effect appeared specific to PAR-2, as curcumin treatment did not significantly alter the expression of the related receptor PAR-1 under the same conditions, using PAR-1 as a relevant control receptor also expressed in these cells and involved in inflammation [53]. Consistent with the inhibition of PAR-2 expression and signaling, curcumin treatment led to a marked reduction in the LPS-induced phosphorylation of ERK1/2, a key downstream effector kinase mediating PAR-2’s pro-proliferative and anti-apoptotic effects. Furthermore, curcumin modulated key regulators of apoptosis, influencing the ratio of anti-apoptotic *Bcl 2* to pro-apoptotic *Bax* proteins and promoting the activation of executioner caspases, thereby countering the survival signals potentially driven by PAR-2. Curcumin also effectively attenuated the LPS-induced elevation of intracellular calcium levels, assessed using the Fluo-4 AM calcium indicator, targeting another critical node in PAR-2 signaling. Functionally, these molecular changes translated into an increased rate of apoptosis in the LPS-stimulated CRC cells following curcumin treatment, as determined by Annexin V/Propidium Iodide flow cytometry. To explore a potential structural basis for these observations, in silico molecular docking was performed using AlphaFold-predicted structures of human PAR-2 and the cbDock2 docking tool. These computational analyses suggested a plausible binding interaction between curcumin and the PAR-2 protein. Collectively, these findings provide novel evidence demonstrating that curcumin can specifically target and downregulate the PAR-2 signaling axis in CRC cells under inflammatory conditions, impacting key downstream pathways involved in proliferation, survival, calcium homeostasis, and apoptosis.

## 2. Materials and Methods

### 2.1. Study Design

This investigation was designed as a cross-sectional in vitro experimental study to characterize the acute effects of curcumin on the modulation of inflammatory mediators in CRC cell lines. The design enables targeted analysis of pro-inflammatory signaling pathways at a specific time point, providing mechanistic insights into curcumin’s bioactivity. This approach eliminates the complexities of longitudinal studies while emphasizing curcumin’s potential as an anti-inflammatory nutraceutical in colorectal carcinogenesis.

### 2.2. Ethics Considerations

All experimental procedures in this study were conducted exclusively in vitro using established, commercially available human colorectal cancer cell lines. The research did not involve the use of animal models, patient-derived specimens, identifiable human biological materials, or human participants.

Accordingly, the study qualifies as minimal risk and is exempt from full Institutional Review Board (IRB) review under the regulations of Mohammed Bin Rashid University of Medicine and Health Sciences (MBRU). Inquiries regarding the ethical review status may be directed to the MBRU IRB at irb@mbru.ac.ae.

No human subjects, identifiable data, or archived clinical samples were accessed; thus, informed consent was not required. The study adhered fully to institutional and international ethical guidelines for in vitro research using non-identifiable biological materials.

### 2.3. Cell Line Selection

**Figure 1:**
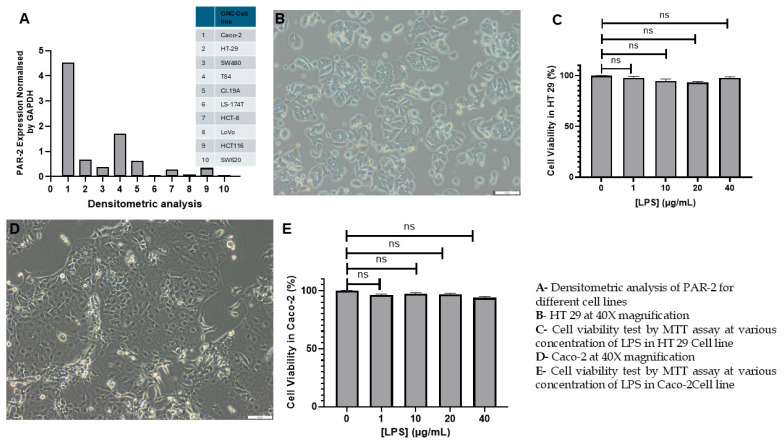
Morphological characterization and LPS cytotoxicity assessment in HT 29 and Caco-2 cells. (**A**) Densitometric analysis of PAR-2 expression across multiple colorectal cancer (CRC) cell lines, normalized to *GAPDH* levels (raw blot data obtained from the study by Darmoul et al. [65]). (**B**) HT 29 cells cultured under standard conditions to 85% confluence exhibit a well-distributed, fibroblast-like morphology, indicative of high proliferative capacity and excellent cell viability. (**C**) MTT assay results for HT 29 cells show no significant reduction in cell viability across LPS concentrations (1, 10, 20, and 40 µg/mL), with viability remaining above 90%. (**D**) Caco-2 cells display their characteristic epithelial clustering and compact colony formation, reflective of their differentiation potential and robust growth under experimental conditions. (**E**) MTT assay results for Caco-2 cells indicate sustained viability exceeding 95% under identical LPS treatments as HT 29. (Note: Bars represent mean ± standard deviation (*n* = 3), compared to untreated controls, indicating no cytotoxic effects. These findings validate the suitability of LPS at tested concentrations for inducing inflammation without compromising cellular integrity. Scale bars: 100 µm, ns: not significant).

To model discrete stages of CRC, HT-29 and Caco-2 cell lines were selected based on their distinct phenotypes and elevated endogenous PAR-2 expression. HT-29, derived from a moderately differentiated grade II adenocarcinoma, exhibits proliferative capacity, EMT potential, and strong inflammatory responsiveness, making it suitable for advanced-stage modeling and PAR-2 signaling studies [66,67]. In contrast, Caco-2, originating from a well-differentiated adenocarcinoma, undergoes spontaneous enterocytic differentiation with apical–basolateral polarity, serving as a robust model of early-stage CRC and epithelial barrier dysfunction [68,69]. Comparative analysis of Northern blot data from Darmoul et al. [64], quantified by densitometry against GAPDH, confirmed that both lines exhibit among the highest PAR-2 expression (Figure 1A).

Other CRC lines were excluded on functional grounds: T84, despite elevated PAR-2, have limited proliferative kinetics and are primarily used for ion transport/barrier assays [70]; SW480 show moderate PAR-2 expression but lack metastatic phenotype and exhibit attenuated cytokine responsiveness [71,72]; HCT-8 display low PAR-2 levels and poor differentiation consistency [73]; and Cl.19A demonstrate transcriptional heterogeneity and variable EMT states that compromise reproducibility [74,75,76]. By contrast, HT-29 and Caco-2 have been extensively validated for epithelial fidelity and reproducibility [77,78], supporting their strategic use here as complementary models of stage-specific CRC and inflammation-driven PAR-2 signaling.

### 2.4. Cell Culture and Treatment

For all experimental procedures, HT 29 (C0009004, AddexBio, San Diego, CA, USA) and Caco-2 (C0009009, AddexBio, San Diego, CA, USA) CRC cells were seeded at a density of 1 × 10^7^ cells per well to ensure standardized culture conditions and optimal cell confluence. This seeding density was determined based on established protocols for in vitro modeling of inflammation-associated signaling pathways in colorectal cancer [79,80]. All assays were performed in biological triplicates (*n* = 3), with each biological replicate comprising multiple technical replicates to control for intra-assay variability. Statistical analyses were conducted on aggregate data to confirm experimental reproducibility and to account for both inter-and intra-experimental variation.

#### 2.4.1. Cell Culture Methodology

HT 29 and Caco-2 colorectal adenocarcinoma cells were cultured under aseptic conditions. Cryopreserved vials were thawed at 37 °C, transferred to pre-warmed medium, and viability was confirmed using Trypan Blue. HT 29 cells were maintained in RPMI 1640 (AL028A, Himedia, Pune, India) with 10% fetal bovine serum (FBS; 16000044, Gibco, Thermo Fisher Scientific, Waltham, MA, USA), 1% penicillin–streptomycin (15140122, Gibco, Thermo Fisher Scientific, Waltham, MA, USA), and 1% L-glutamine, while Caco-2 cells were cultured in high-glucose DMEM (11965092, Gibco, Thermo Fisher Scientific, Waltham, MA, USA) with 10% FBS, 1% penicillin–streptomycin, and 1% non-essential amino acids. After centrifugation (200× *g*, 5 min), cells were seeded at 1 × 10^7^ cells/mL in T-75 flasks and incubated at 37 °C with 5% CO_2_. Cultures were monitored daily, with medium refreshed every 48–72 h and subcultured at 70–80% confluence to maintain logarithmic growth.

#### 2.4.2. Subculturing Cryopreservation of HT 29 and Caco-2 Cells

At 70–80% confluence, cells were rinsed with PBS (pH 7.4) (10010023, Gibco, Thermo Fisher Scientific, Waltham, MA, USA) and detached using 0.25% Trypsin–EDTA (15400054, Gibco, Thermo Fisher Scientific, Waltham, MA, USA) at 37 °C for 2–4 min, with detachment monitored microscopically to avoid over-trypsinization. Trypsinization was neutralized with complete medium, and cells were centrifuged at 200× *g* for 5 min. Pellets were resuspended in fresh medium, reseeded at a 1:3 ratio into T-75 flasks, and incubated at 37 °C with 5% CO_2_.

For cryopreservation, cells at 70–80% confluence were harvested similarly and resuspended in freezing medium (complete medium + 5% (dimethyl sulfoxide) DMSO) at 1 × 10^6^ cells/mL. One-milliliter aliquots were placed in cryovials (5000-0020, Gibco, Thermo Fisher Scientific, Waltham, MA, USA) and frozen at a controlled rate of −1 °C/min (Mr. Frosty™, Thermo Fisher Scientific, Waltham, MA, USA) before storage in liquid nitrogen (−196 °C), ensuring high post-thaw viability and phenotypic stability.

### 2.5. Assessment of Cytotoxicity Using the MTT Assay

The cytotoxicity of lipopolysaccharide (LPS; 00-4976-03, Thermo Fisher Scientific, Waltham, MA, USA) was assessed in HT 29 and Caco-2 cells using the 3-(4,5-dimethylthiazol-2-yl)-2,5-diphenyltetrazolium bromide (MTT) assay as per standard protocols [81]. The assay was performed using the commercially available MTT reagent (M6494, Thermo Fisher Scientific, Waltham, MA, USA), which quantifies mitochondrial-dependent metabolic activity as a surrogate marker of cell viability. Cells were exposed to graded LPS concentrations, and mitochondrial activity was quantified by spectrophotometric measurement of formazan at 570 nm. Viability was expressed relative to untreated controls, enabling the selection of non-cytotoxic LPS concentrations for subsequent inflammation induction.

### 2.6. Prepration of Curcumin Stock Solution

Curcumin (≥98% purity, HY-N0005, MedChem Express, Monmouth Junction, NJ, USA) was accurately weighed and dissolved in anhydrous (DMSO; D12345, Thermo Fisher Scientific, Waltham, MA, USA) to prepare a 50 mM stock solution. Complete dissolution was ensured by gentle vortexing and brief sonication under light-protected conditions. The stock solution was aliquoted into sterile amber microtubes to prevent photodegradation and stored at −20 °C to avoid repeated freeze–thaw cycles. For all treatments, the stock was freshly diluted in pre-warmed complete culture medium to achieve final curcumin concentrations of 50 μM and 100 μM for both HT 29 and Caco-2 cells. The final DMSO concentration did not exceed 0.2% (*v*/*v*) in any experimental or vehicle control groups, and parallel solvent control (0.2% DMSO) treatments were included to rule out solvent-related effects.

### 2.7. Induction of Inflammation in HT 29 and Caco-2 Cells Using LPS and Curcumin Treatment

To establish an in vitro inflammatory model relevant to colorectal carcinogenesis, HT-29 and Caco-2 cells were exposed to LPS (10 µg/mL), a concentration optimized in prior studies and widely used for TLR4 activation in epithelial systems [53,54,81]. At this dose, LPS activates NF-κB and induces TNF-α without nonspecific cytotoxicity [52]. LPS was deliberately selected over direct PAR-2 agonists because it better emulates the CRC inflammatory milieu: TLR4 stimulation enhances endogenous protease expression (tryptase-β, neutrophil elastase, MMP-9) [82,83,84], upregulates PAR-2 itself [85,86], and recapitulates bidirectional TLR4–PAR-2 crosstalk [77,82]. By contrast, trypsin exhibits proteolytic promiscuity (PAR-1,-3,-4 activation), receptor desensitization, and rapid inactivation by protease inhibitors [87,88,89,90], while synthetic PAR2-APs such as SLIGRL-NH_2_ lack microbial/cytokine context, induce off-target Mrgpr signaling, are metabolically unstable, and fail to trigger canonical NF-κB/TNF-α responses [91,92,93]. In vivo data further demonstrate that PAR-2 blockade mitigates LPS-induced inflammation, underscoring their synergy [94].

Curcumin was applied 24 h post-LPS at 50 and 100 µM, doses repeatedly shown to exert antiproliferative, pro-apoptotic and anti-inflammatory effects in CRC lines, including G2/M arrest, Bcl-2 modulation, caspase-3/-12 activation, p53 Ser15 phosphorylation, and inhibition of ERK/mTOR signaling [41,95,96,97]. These concentrations also suppress EGR-1/Elk-1 [96,98], mobilize intracellular Ca^2+^, and induce ROS-mediated apoptosis [42,99,100]. Although systemic free curcumin rarely exceeds 20 nM, colonic tissue levels reach high micromolar to millimolar ranges (≤131 µM in biopsies; ≤12,937 µM in rectal tumors) [101,102,103], supported by luminal accumulation from poor absorption and fecal excretion [104,105]. Thus, 50–100 µM approximates localized gastrointestinal exposure, providing a physiologically relevant framework for dissecting curcumin’s modulatory effects on TLR4 → PAR-2 signaling in inflammation-driven CRC.

### 2.8. RNA Extraction and cDNA Synthesis

Total RNA was extracted from both control and inflammatory (LPS-treated) HT 29 and Caco-2 cells before and after treatment with curcumin at concentrations of 50 and 100 µM, using the Total RNA Isolation Kit (MB13402, NZYTech, Lisbon, Portugal). In parallel experiments designed to assess the ERK–TNF-α regulatory axis, cells were treated with the selective ERK inhibitor SCH772984 at a final concentration of 10 µM prior to RNA extraction. The purity and integrity of the isolated RNA were carefully assessed using a Nanodrop Spectrophotometer (Thermo Scientific, Waltham, MA, USA), ensuring consistent RNA quality across all biological replicates. Only samples exhibiting A260/A280 ratios within the range of 1.8 to 2.0 were deemed suitable for subsequent analysis.

The high-quality RNA samples were then reverse-transcribed into complementary DNA (cDNA) using the First-Strand cDNA Synthesis Kit (NP100042, OriGene, Heidelberg, Germany), strictly following the manufacturer’s protocol [106]. The resulting cDNA was utilized as a template for real-time quantitative PCR (qPCR) to assess the expression of a panel of target genes: *PAR-1*, *PAR-2*, *TNF-α*, *Bcl 2*, *Bax*, *caspase-3*, *caspase-8*, *ERK1*, *ERK2*, *pERK* and *GAPDH*, providing mechanistic insights into curcumin’s modulatory effect on inflammation and apoptosis under simulated pro-inflammatory conditions.

#### Real-Time PCR for Quantification (qPCR)

qPCR was performed with stringent quality control using the QuantStudio 5 Flex Real-Time PCR System (Applied Biosystems, Waltham, MA, USA) to measure mRNA expression of the selected targets: *PAR-1*, *PAR-2*, *TNF-α*, *Bcl 2*, *Bax*, *caspase-3*, *caspase-8*, *ERK1*, *ERK2*, *pERK* and *GAPDH*. SYBR Green chemistry was employed for real-time fluorescence detection due to its high sensitivity and reproducibility in double-stranded DNA quantification.

All primers were designed using OriGene’s proprietary algorithm, specifically optimized for sequence specificity, GC content, melting temperature, and minimized secondary structure formation. The primer sequences underwent comprehensive in silico validation against the human genome using BLAST (version BLAST+ 2.13.0) to confirm high target specificity and absence of off-target binding. Thermodynamic features such as annealing temperature, hairpin structure, and dimer formation potential were evaluated using Primer-BLAST and OligoAnalyzer to ensure primer integrity and robust amplification performance.

Primer quality was further validated through E-value and Bit Score metrics, with all primers showing E-values of 0.0, reflecting exact sequence alignment with their respective targets. Bit Scores ranged from 2374 for *GAPDH* to 2861 for PAR-2, indicative of optimal primer-template affinity. These results are compiled in Table 1 and collectively underscore the stringent bioinformatic quality control underpinning primer selection.

qPCR reactions were performed in technical triplicates with no-template controls to exclude contamination. Cycling comprised an initial 95 °C denaturation, 40 cycles of denaturation/annealing/extension, and a terminal melt-curve for specificity assessment. Relative quantification used the 2^−ΔΔCt^ method with GAPDH as housekeeping control; ΔCt values were normalized to GAPDH and ΔΔCt values calculated against untreated controls (set to 1.0). Amplification efficiency was validated by standard curves from serial cDNA dilutions, with all primer sets demonstrating 90–110% efficiency and R^2^ > 0.99. This protocol ensured robust, reproducible quantification of curcumin-induced transcriptional changes under inflammatory stress.

### 2.9. Western Blot Analysis: Comprehensive Evaluation of PAR-2-Associated Signaling Axis and Apoptotic Pathways

To interrogate the mechanistic effects of curcumin on key inflammatory and apoptotic signaling cascades in CRC, Western blotting was employed to quantify the expression levels of several proteins central to PAR-mediated signaling and downstream apoptotic pathways in HT 29 and Caco-2 cell lines. The protein targets analyzed included PAR-2 (6976T, Cell Signaling, Danvers, MA, USA), PAR-1 (79109T, Cell Signaling, Danvers, MA, USA), total ERK1/2 (83533-1-RR, Proteintech, Minneapolis, MN, USA), phosphorylated ERK1/2 (Thr202/Tyr204) (20582-1-AP, Proteintech, Minneapolis, MN, USA), cleaved *caspase-3* (25128-1-AP, Proteintech, Minneapolis, MN, USA), pro-*caspase-8* (13423-1-AP, Proteintech, Minneapolis, MN, USA), Cleaved *Caspase-8* (9496T, Cell Signaling, Danvers, MA, USA) B-cell lymphoma 2 (*Bcl 2*, 3498T, Cell Signaling, Danvers, MA, USA), and *Bcl 2*-associated X protein (*Bax*, 50599-2-Ig, Proteintech, Minneapolis, MN, USA), with *GAPDH* (2118T, Cell Signaling, Danvers, MA, USA) used as a housekeeping control.

#### 2.9.1. Rationale for the Selection of Protease-Activated Receptors and Biochemical Markers in This Study

Protease-Activated Receptors: PAR-2 and PAR-1. PAR-2 was the principal focus of this investigation, given its established role in inflammation-driven carcinogenesis in CRC. Activation of PAR-2 by trypsin-like serine proteases initiates downstream signaling via Gα_q and β-arrestin pathways, culminating in phosphorylation of ERK1/2, increased intracellular calcium, and modulation of pro-survival and apoptotic machinery [57]. To determine the specificity of curcumin’s regulatory effects on PAR-2, we included PAR-1 as a comparator control. Although PAR-3 exhibited slightly greater sequence identity with PAR-2 than PAR-1 (Refer below), it primarily serves as a cofactor in PAR-1/PAR-4 signaling complexes rather than an autonomous signaling receptor [107].

To support the selection of PAR-1, we conducted pairwise sequen.ce alignments using Clustal Omega (v1.2.4) [108] and visualized the alignments via Jalview (v2.11.4.1) [109]. The results revealed the following percentage sequence identities with human PAR-2: PAR-1 (34.25%), PAR-3 (34.90%), and PAR-4 (32.15%). Despite the marginally higher sequence identity of PAR-3, its limited signaling autonomy and predominant function as a dimerization partner exclude it as a reliable standalone comparator. PAR-4, though expressed in CRC tissues, was excluded due to its less consistent expression in normal mucosa and weaker oncogenic signaling footprint relative to PAR-1 and PAR-2 [110]. PAR-1 was thus selected as the most appropriate internal control, enabling us to distinguish whether curcumin’s modulatory effects were exclusive to PAR-2 or broadly applicable across structurally similar GPCRs.

MAPK Pathway: Total and Phospho-ERK1/2—ERK1/2 (extracellular signal-regulated kinases 1 and 2) represent terminal effectors of the MAPK cascade. Upon PAR-2 activation, G-protein-dependent signaling and β-arrestin-mediated scaffolding synergistically activate Raf/MEK/ERK, driving transcriptional programs that promote proliferation and inflammation. Phosphorylation of ERK1/2 at Thr202/Tyr204 is a canonical readout of pathway activation [111]. Since curcumin is known to inhibit ERK phosphorylation via disruption of upstream kinases and transcriptional regulators (e.g., Egr-1) [25], inclusion of both total and phospho-ERK was necessary to determine the specific regulatory point of intervention.

Apoptotic Markers: *Caspase-3*, *Caspase-8*, *Bcl 2*, and *Bax*. We further analyzed the intrinsic and extrinsic apoptotic axes via Western blotting for cleaved *caspase-3* (executioner caspase) [112], cleaved *caspase-8* (initiator caspase in death receptor pathway) [113], *Bcl 2* (anti-apoptotic protein) [114], and *Bax* (pro-apoptotic pore-forming protein) [115]. In addition, cleaved *caspase-8* was specifically examined to provide stronger evidence of extrinsic apoptotic activation, given its central role in FasL/TNF-α-mediated signaling [116]. This decision was based on the mechanistic evidence that curcumin at 50–100 μM induces apoptosis in CRC cells by triggering mitochondrial outer membrane permeabilization (MOMP) and death receptor signaling, both of which converge on *caspase-3* activation [34]. Curcumin downregulates *Bcl 2* and upregulates *Bax*, shifting the *Bcl 2*/*Bax* ratio toward apoptosis. *Caspase-8* activation, meanwhile, serves as an upstream marker of FasL/TNFα-induced extrinsic apoptosis, further linking curcumin’s anti-inflammatory effects to apoptotic outcomes [117].

#### 2.9.2. Protein Extraction and Quantification

The protein extraction and blotting procedure replicated our previously validated protocol used in the oleocanthal study to ensure methodological consistency [54]. Briefly, HT 29 and Caco-2 cells were subjected to LPS-induced inflammatory priming followed by curcumin treatment at 50 and 150 µM concentrations. Total cellular proteins were extracted using ice-cold lysis buffer (0.5% SDS, 50 mM Tris-HCl, pH 7.4) containing protease (78429, Thermo Fisher Scientific, Waltham, MA, USA) and phosphatase inhibitors (78420, Thermo Fisher Scientific, Waltham, MA, USA). Supernatants obtained after centrifugation at 12,000× *g* for 15 min at 4 °C were subjected to protein quantification using the BCA assay (A55865, Thermo Fisher Scientific, Waltham, MA, USA). Standardized amounts (20 µg per lane) were resolved by SDS-PAGE and transferred onto 0.45-µm nitrocellulose membranes (1620115, Bio-Rad, Mississauga, ON, Canada).

#### 2.9.3. Antibody Incubation and Detection

Membranes were blocked with 3% BSA in Tris-buffered saline (TBS) for 1 h at 4 °C, followed by overnight incubation at 4 °C with primary antibodies against PAR-1, PAR-2, ERK1/2, p-ERK1/2 (Thr202/Tyr204), *caspase-3*, *caspase-8*, cleaved caspase-8, *Bcl 2*, and *Bax* (all from Santa Cruz Biotechnology, diluted 1:1000 in SuperBlock/TTBS). After washing, membranes were incubated with HRP-conjugated secondary antibodies (1:2000 dilution) and visualized using SuperSignal ULTRA Chemiluminescent Substrate (Pierce). Films were developed using Kodak Biomax (GE Healthcare, Chikago, IL, USA).

#### 2.9.4. Densitometry and Statistical Analysis

Band intensities were quantified using ImageJ (v1.54, (National Institute of Health) NIH) [118], and normalized to *GAPDH*, whose stable expression was verified across conditions. Corrected intensities were calculated using the integrated density function with background subtraction. Fold changes were calculated relative to untreated controls. Data represent means ± SEM from at least three independent experiments. Statistical analyses were performed using one-way ANOVA with Tukey’s post hoc test [119] using GraphPad Prism v9.0, with *p* < 0.05 considered significant.

### 2.10. Assessment of TNF-α Secretion via Quantitative ELISA Following Curcumin Treatment

To evaluate the modulatory effects of curcumin on inflammatory cytokine output, the concentration of TNF-α was quantified in the conditioned media collected from both treated and untreated HT 29 and Caco-2 colorectal carcinoma cell lines. Post-treatment, culture supernatants were harvested and subjected to centrifugation at 3000 rpm for 10 min at 4 °C to eliminate residual cellular particulates and ensure sample clarity. The clarified supernatants were assayed for TNF-α using a commercially available enzyme-linked immunosorbent assay (ELISA) kit (ab181421, Abcam, Cambridge, MA, USA), with all steps performed strictly in accordance with the manufacturer’s instructions. In parallel experiments, cells were treated with the selective ERK inhibitor SCH772984 at a final concentration of 10 µM to assess the impact of ERK pathway inhibition on TNF-α expression. The assay employed pre-coated 96-well plates with immobilized anti-TNF-α monoclonal antibodies to ensure specificity. Absorbance was measured at 450 nm, with background correction performed at 620 nm, using a Hidex microplate reader. This methodological approach enabled sensitive and reproducible quantification of TNF-α secretion, thus serving as a critical readout for assessing the anti-inflammatory efficacy of curcumin at specified concentrations in colorectal cancer cell line models.

### 2.11. Dual Fluorescence Staining and Quantification of Apoptotic Cells Using Acridine Orange/Ethidium Bromide (AO/EtBr)

Apoptosis was assessed using Acridine Orange (AO; A1301, Thermo Fisher Scientific, Waltham, MA, USA) and Ethidium Bromide (EtBr; 15585011, Thermo Fisher Scientific, Waltham, MA, USA) dual fluorescent staining, which discriminates viable, early apoptotic, and late apoptotic/necrotic cells by membrane integrity and nuclear morphology [120]. HT-29 and Caco-2 cells (2 × 10^5^/well, 6-well plates, 2 mL DMEM) were cultured to 70–80% confluence at 37 °C/5% CO_2_, treated with LPS (10 µg/mL, 24 h), and subsequently exposed to curcumin (100 µM, 24 h). Controls included untreated, LPS-only, and LPS + curcumin groups. After treatment, cells were washed twice with PBS and incubated for 10 min in the dark with 1 mL dual staining solution (AO and EtBr, each 100 µg/mL in PBS). AO intercalated into DNA of all cells (green fluorescence), while EtBr entered only membrane-compromised cells (orange/red fluorescence). Stained cells were immediately visualized on a Leica DMi8 fluorescence microscope (excitation 488 nm; emission 515–620 nm), and representative CCD images were captured to document viable, apoptotic, and necrotic populations.

Cell populations were analyzed based on fluorescence characteristics: viable cells fluoresced uniformly green; early apoptotic cells exhibited bright green nuclei with condensed chromatin; and late apoptotic or necrotic cells showed orange to red fluorescence due to EtBr penetration. Manual quantification was performed by blinded observers, and automated validation was conducted using ImageJ software 1.54p (NIH, MD, USA) with the “Cell Counter” and “Analyze Particles” tools. A minimum of 500 cells per condition were analyzed across replicate fields. The apoptotic index was calculated using the formula:

*Apoptotic Index* (%) = (*Number of apoptotic cells*/*Total number of cells*) × 100

Statistical analysis was performed using GraphPad Prism 9.0 (GraphPad Software, La Jolla, CA, USA). Data were expressed as mean ± standard error of the mean (SEM) from three independent experiments (*n* = 3). One-way ANOVA followed by Tukey’s post hoc test was applied to determine statistical significance, with *p* < 0.05 considered significant.

### 2.12. Annexin V-FITC and Propidium Iodide (PI) Dual Staining for Apoptosis Detection

Apoptosis was quantified by Annexin V-FITC/PI dual staining (BMS500FI-300, Thermo Fisher Scientific, Waltham, MA, USA) followed by flow cytometry, distinguishing viable, early apoptotic, late apoptotic, and necrotic cells via phosphatidylserine externalization and membrane integrity. HT-29 and Caco-2 cells (3 × 10^5^/well, 6-well plates) were cultured to 70–80% confluence, primed with LPS (10 µg/mL, 24 h), and treated with curcumin (100 µM, 24 h). Adherent and floating cells were harvested by trypsinization, centrifuged (300× *g*, 5 min, 4 °C), washed twice in PBS, and resuspended in 100 µL 1× binding buffer. Staining was performed with 5 µL Annexin V-FITC and 5 µL PI (Annexin V-FITC Apoptosis Detection Kit, BioLegend, San Diego, CA, USA) for 15 min at room temperature in the dark, followed by dilution in 400 µL binding buffer. Samples were analyzed immediately on a BD FACSCanto II flow cytometer (BD Biosciences, San Francisco, CA, USA) with excitation/emission of 488/530 nm (FITC) and 488/617 nm (PI). Compensation and quadrant gates were established using unstained and single-stained controls. A minimum of 10,000 gated events/sample were acquired, and data were processed in FlowJo v10.9.1 (BD Biosciences). Results were expressed as mean ± SEM from *n* = 3 independent replicates. Statistical analyses were performed in GraphPad Prism 9.0 (GraphPad Software, San Diego, CA, USA) using one-way ANOVA with Tukey’s post hoc test; *p* < 0.05 was considered significant.

### 2.13. Calcium Signaling Assay

Intracellular calcium signaling regulates proliferation, apoptosis, and inflammation, and is mechanistically coupled to PAR-2 activation [121]. In CRC, PAR-2 stimulation activates phospholipase C (PLC), generating InsP_3_ and mobilizing Ca^2+^ from ER stores, with the resultant surge potentiating NF-κB–driven pro-inflammatory pathways [122]. To examine curcumin’s effect on Ca^2+^ flux under inflammatory stress, HT-29 and Caco-2 cells were cultured to 70–80% confluence in 35 mm dishes (2 mL DMEM). Cells were washed twice with pre-warmed PBS and loaded with 2 µM Fluo-4 AM (F14217, Thermo Fisher Scientific, Waltham, MA, USA) in HBSS + 1 mM CaCl*_2_* for 30 min at 37 °C, followed by 30 min de-esterification at room temperature. Inflammation was induced with LPS (10 µg/mL), after which cells were treated with curcumin (50 or 100 µM, 24 h), consistent with parallel apoptosis and anti-inflammatory assays. Real-time Ca^2+^ imaging was performed using a Leica (Wetzlar, Germany) DMi8 fluorescence microscope (excitation 488 nm, emission 516 nm), and fluorescence intensity was quantified under live-cell conditions. This assay enabled high-resolution monitoring of Ca^2+^ dynamics and clarified how curcumin modulates PAR-2–dependent calcium signaling during inflammation-driven CRC.

### 2.14. Computational Modeling of PAR-2

The complete amino acid sequence of human PAR-2 was retrieved from the UniProt Knowledgebase [123], (Accession ID: P55085) [86] and formatted in FASTA for structural modeling. Comprising 397 amino acids, the sequence was input into AlphaFold v2.3, an advanced deep learning-based framework known for achieving near-experimental accuracy in protein structure prediction [124]. AlphaFold combines multi-sequence alignment (MSA), deep residual neural networks, and physical constraints to reconstruct high-fidelity three-dimensional structures. To generate the evolutionary profile, homology searches were performed using MMseqs2 across UniRef90, MGnify, and PDB70 databases [125]. No crystallographic templates were applied during modeling to avoid structural bias.

The prediction was executed in monomer mode, producing five structural models ranked by internal confidence metrics. These include the Predicted Local Distance Difference Test (pLDDT), Predicted Aligned Error (PAE), and per-residue Interatomic Distance Difference Test (pIDDT) scores, each serving as an independent indicator of structural reliability. The model with the highest global pLDDT was selected for downstream analysis.

Per-residue reliability was first evaluated using pLDDT scores, ranging from 0 to 100. Scores above 90 were indicative of high-confidence atomic positioning, values between 70 and 90 suggested moderate confidence, and scores below 50 marked structurally disordered or flexible segments. These scores were extracted from AlphaFold’s raw output and visualized using Matplotlib (v2.3.0) [124,126]. PAE matrices were then employed to evaluate pairwise residue alignment uncertainty, with values < 5 Å representing structural rigidity and those > 15 Å denoting high flexibility, especially within loop regions. These matrices were visualized as heatmaps to identify inter-domain dynamics, particularly across extracellular and intracellular loops [127].

To complement these assessments, pIDDT scores were analyzed to quantify per-residue structural stability, independent of domain-wide conformational constraints [128]. Residues with pIDDT scores > 80 were categorized as structurally stable, whereas those < 60 were considered conformationally labile. The pIDDT profiles were plotted to correlate local flexibility with predicted structural disorder.

For three-dimensional structural inspection and domain annotation, ChimeraX v1.4 was employed [129]. The AlphaFold-predicted model was visualized in cartoon representation, with explicit demarcation of transmembrane helices, intracellular and extracellular loops. Secondary structure elements—α-helices, β-sheets, and loops—were color-mapped for visual clarity, although this representation was not used for quantitative assessment.

To ensure comprehensive model evaluation, a set of validation figures was generated. These included: a sequence coverage map derived from the MSA outputs to indicate evolutionary conservation [130]; a pLDDT-mapped 3D structure to delineate high-confidence core regions from low-confidence peripheries; a PAE heatmap to locate flexible domains; and a pIDDT line graph for visualizing per-residue stability.

Taken together, this integrative multi-metric validation enabled a rigorous assessment of PAR-2 structural reliability, delineating high-confidence transmembrane domains from dynamic loop regions—features essential for understanding GPCR topology, conformational plasticity, and ligand-binding behavior.

### 2.15. Molecular Docking of Curcumin with AlphaFold-Predicted PAR-2 Structure

Molecular docking simulations to assess the interaction of curcumin with PAR-2 were performed using CB-Dock2 [131,132], a blind docking platform that integrates curvature-based binding site detection with AutoDock Vina-driven flexible docking algorithms [133]. This system further incorporates a homologous template-guided docking refinement protocol, thus offering a synergistic combination of geometric cavity recognition and evolutionary ligand-pose prediction. The objective of this docking study was to elucidate the potential molecular interactions between curcumin and the transmembrane domain of PAR-2, a G-protein-coupled receptor implicated in pro-inflammatory signaling pathways relevant to colorectal cancer biology.

The three-dimensional structure of PAR-2 used for docking was obtained from the AlphaFold v2.3 prediction pipeline, with the highest-confidence model selected based on global pLDDT scores and supporting structural metrics. Given PAR-2’s classification as a Class A GPCR with a seven-transmembrane helical topology, the receptor structure was pre-processed to retain proper helical orientation, extracellular loop integrity, and intracellular conformational domains.

The molecular structure of curcumin was retrieved from the MedChem database in its lowest energy conformation and subsequently converted to PDBQT format to define torsional flexibility for docking simulations. All stereochemical configurations and tautomeric states were preserved during ligand preparation to ensure accurate modeling of curcumin’s interaction potential.

CB-Dock2 follows a hierarchical computational framework, beginning with curvature-based pocket prediction to identify topographically accessible binding cavities on the protein surface. In parallel, the server performs a template-based retrieval using FP2 fingerprint similarity indices (FP2 ≥ 0.4) to scan its internal repository of ligand–protein complexes. If a template ligand is identified with ≥40% sequence identity at the predicted binding pocket and a pocket RMSD ≤ 4 Å, the FitDock refinement module is triggered. This allows template-guided pose optimization prior to docking, enhancing the accuracy of ligand placement.

Subsequently, AutoDock Vina was employed for flexible ligand docking within each identified cavity, using an empirical scoring function to estimate the free energy of binding (ΔG, kcal/mol). In cases where template ligands were identified, curcumin was aligned to the reference ligand’s binding pose using FitDock, followed by refinement of dihedral angles and conformational sampling within the pocket constraints.

The docking simulations generated multiple binding poses for curcumin, each ranked by binding energy and pose confidence (PC-score). The final pose was selected based on the lowest predicted binding energy and the highest confidence metric, indicating optimal spatial complementarity and stability of the ligand–receptor complex.

For downstream analysis, the selected docking conformation was visualized using PyMOL and Discovery Studio Visualizer, enabling detailed mapping of key molecular interactions. Hydrogen bonds, π–π stacking, hydrophobic contacts, and electrostatic interactions between curcumin and critical transmembrane domain residues of PAR-2 were identified. This allowed the characterization of curcumin’s potential binding mechanism and receptor engagement, contributing to mechanistic insights into its modulatory effects on PAR-2-mediated inflammatory signaling.

## 3. Results

### 3.1. Assessment of LPS-Induced Cytotoxicity and Establishment of an Inflammatory CRC Model in HT 29 and Caco-2 Cell Lines

The morphological and viability analyses of HT 29 and Caco-2 cells following LPS treatment were conducted to establish a reliable in vitro model of inflammation-driven CRC. For justification of cell line selection and their established relevance to inflammation-linked colorectal pathology, the reader is referred to the Methods section.

Microscopic evaluation of untreated HT 29 cells (Figure 1B) revealed an adherent monolayer exhibiting characteristic elongated, fibroblast-like morphology with planar organization. Cells were distributed uniformly, with no discernible evidence of detachment, cytoplasmic blebbing, or vacuolization. These features reflected an intact cytoskeletal framework and confirmed the proliferative competency of HT 29 under basal conditions. The phase-contrast imaging further demonstrated minimal intercellular spacing, suggesting robust tight junction formation and effective cell–cell adhesion. Such a phenotype is indicative of an epithelial-to-mesenchymal hybrid state, often retained in CRC lines with migratory potential.

In parallel, untreated Caco-2 cells (Figure 1D) maintained their classical epithelial morphology, forming tightly packed cobblestone-like clusters consistent with spontaneous enterocyte differentiation. High nuclear-to-cytoplasmic ratio and clearly demarcated cellular borders were observed, signifying a polarized architecture essential for barrier function modeling. The absence of membrane blebbing or perinuclear clearing reinforced their viability and functional differentiation status.

To assess the cytotoxic potential of LPS and delineate a working dose that elicits inflammation without impairing viability, both cell lines were subjected to increasing concentrations of LPS (1, 10, 20, and 40 µg/mL), and metabolic viability was quantified using the MTT assay. As depicted in Figure 1C, HT 29 cells retained over 90% viability across all LPS concentrations. There was no observable dose-dependent decline in formazan absorbance, indicating that mitochondrial dehydrogenase activity—and, by extension, cellular metabolic competence—was largely preserved despite the inflammatory challenge.

Similarly, Caco-2 cells exhibited minimal deviation from untreated controls across the entire concentration range (Figure 1E), with viability consistently exceeding 95%. The maintenance of high metabolic activity confirmed that LPS-induced signaling did not exert deleterious effects on cell health. These findings validate the suitability of LPS at tested concentrations for inducing inflammation without compromising cellular integrity. Importantly, neither morphological anomalies nor viability losses were detected at 10 µg/mL, the dose selected for downstream inflammatory stimulation. This concentration was chosen as it aligns with established thresholds for TLR4-mediated pro-inflammatory activation without perturbing cellular integrity.

While the direct cytokine quantification is not represented in this figure, ELISA investigations confirmed the efficacy of 10 µg/mL LPS in triggering TNF-α secretion, with a 58.47% increase in HT 29 and a 51.39% increase in Caco-2 cells over untreated conditions. This cytokine induction substantiates the activation of canonical inflammatory pathways downstream of LPS engagement and confirms the establishment of a non-cytotoxic, yet pro-inflammatory, CRC model suitable for investigating anti-inflammatory interventions and molecular modulators of inflammation-driven oncogenesis.

These findings collectively underscore the suitability of HT 29 and Caco-2 cells as stable and responsive platforms for modeling chronic inflammation in colorectal carcinogenesis. Their preserved morphology sustained metabolic activity, and reproducible cytokine output in response to LPS stimulation validate their role as foundational models for subsequent mechanistic and pharmacological studies.

### 3.2. Curcumin Downregulates PAR-2 Expression at the Transcriptional and Translational Level in HT 29 and Caco-2 Cells

To investigate the regulatory effect of curcumin on PAR-2 expression under inflammatory conditions, we examined both protein and mRNA levels in HT 29 and Caco-2 CRC cell lines following treatment with 50 µM and 100 µM curcumin, after LPS-induced stimulation (10 µg/mL), as justified in the Section 1 and Section Real-Time PCR for Quantification (qPCR).

**Figure 2:**
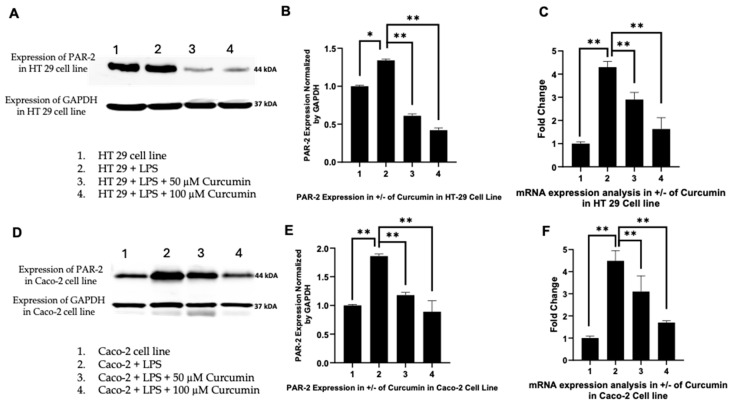
Curcumin attenuates LPS-induced PAR-2 expression at both protein and mRNA levels in HT 29 and Caco-2 cells. (**A**) Representative Western blot showing protein expression levels of Protease-Activated Receptor-2 (PAR-2) in HT 29 cells under four experimental conditions: untreated control (*Lane 1*), LPS-treated (10 µg/mL for 24 h; *Lane 2*), LPS + 50 µM curcumin (*Lane 3*), and LPS + 100 µM curcumin (*Lane 4*). *GAPDH* served as the loading control. A specific PAR-2 band was detected at ~44 kDa, corresponding to the unglycosylated core protein; (**B**) Densitometric quantification of PAR-2 protein expression in HT 29 cells normalized to *GAPDH* and expressed as relative fold-change compared to the untreated control (*Lane 1*). Data represent mean ± SEM from three independent experiments; (**C**) Quantitative real-time PCR (qPCR) analysis of PAR-2 mRNA expression in HT 29 cells under identical treatment conditions as panel A. Results are expressed as fold-change relative to the untreated control using the 2^−ΔΔCt^ method, with *GAPDH* as the housekeeping gene; (**D**) Representative Western blot of PAR-2 expression in Caco-2 cells under corresponding experimental conditions as in panel A. Lanes 1 to 4 represent untreated, LPS-treated, and LPS + curcumin (50 µM and 100 µM) conditions, respectively; (**E**) Densitometric analysis of PAR-2 protein expression in Caco-2 cells, normalized to *GAPDH*, and expressed as relative fold-change compared to the control; (**F**) qPCR analysis of PAR-2 mRNA expression in Caco-2 cells, showing downregulation upon curcumin treatment. (Note: All protein and mRNA data are representative of triplicate experiments (*n* = 3). Statistical analysis was performed using one-way ANOVA followed by Tukey’s post hoc test. * *p* < 0.05; ** *p* < 0.01).

In HT 29 cells (Figure 2A–C), Western blot analysis revealed a marked attenuation of PAR-2 protein expression following curcumin treatment. Compared to LPS-stimulated controls, which displayed a robust upregulation of PAR-2, curcumin at 50 µM reduced protein expression visibly, with a further pronounced suppression at 100 µM. Densitometric quantification (Figure 2B), normalized to *GAPDH*, substantiated this observation: a statistically significant reduction in PAR-2 band intensity was evident at both curcumin concentrations (*p* < 0.05), with a ~42.8% decrease at 50 µM and ~68.3% reduction at 100 µM compared to LPS-only controls.

This trend was mirrored at the mRNA level, as assessed by quantitative real-time PCR (Figure 2C). LPS treatment led to a significant induction of PAR-2 transcript levels in HT 29 cells relative to untreated controls. However, curcumin administration significantly blunted this effect in a dose-dependent manner. A ~1.7-fold suppression of LPS-induced PAR-2 mRNA expression was observed at 50 µM, which further deepened to ~2.6-fold at 100 µM (*p* < 0.01). These findings collectively demonstrate that curcumin downregulates PAR-2 expression at the transcriptional level, likely contributing to reduced protein synthesis and downstream receptor signaling.

Parallel experiments in Caco-2 cells (Figure 2D–F) produced concordant results. Immunoblot analysis (Figure 2D) showed that curcumin treatment substantially reduced PAR-2 protein levels post-LPS stimulation, with densitometric quantification (Figure 2E) confirming a dose-dependent attenuation. Specifically, PAR-2 protein levels were reduced by ~39.4% at 50 µM and ~63.2% at 100 µM curcumin (relative to LPS-only treatment, *p* < 0.05). The transcript-level data (Figure 2F) also revealed a significant repression of PAR-2 mRNA, with a ~1.6-fold and ~2.4-fold decrease at 50 µM and 100 µM curcumin, respectively (*p* < 0.01). These findings indicate that curcumin exerts consistent inhibitory effects on PAR-2 expression across distinct CRC cellular phenotypes.

Together, these results robustly establish that curcumin effectively counteracts the LPS-induced upregulation of PAR-2 in CRC cells. This suppression at both protein and mRNA levels reinforces curcumin’s potential as an anti-inflammatory agent targeting PAR-2-mediated signaling in the CRC inflammatory microenvironment. These results also validate PAR-2 as a pharmacologically responsive target modulated by naturally occurring bioactives in epithelial tumor models.

### 3.3. Curcumin Does Not Significantly Alter PAR-1 Expression in LPS-Stimulated HT 29 and Caco-2 Cells

To determine whether the modulatory effects of curcumin observed on PAR-2 expression were specific or extended to other members of the PAR family, the expression of PAR-1 was analyzed in both HT 29 and Caco-2 colorectal cancer cell lines. As explicated in Methodology (refer above), PAR-1 was selected as a comparator based on its structural and functional homology with PAR-2, while being activated by distinct proteolytic stimuli (thrombin rather than trypsin-like proteases). The comparative analysis included evaluation at both the protein and mRNA levels following treatment with 50 µM and 100 µM curcumin under LPS-induced inflammatory conditions (10 µg/mL, 24 h).

**Figure 3:**
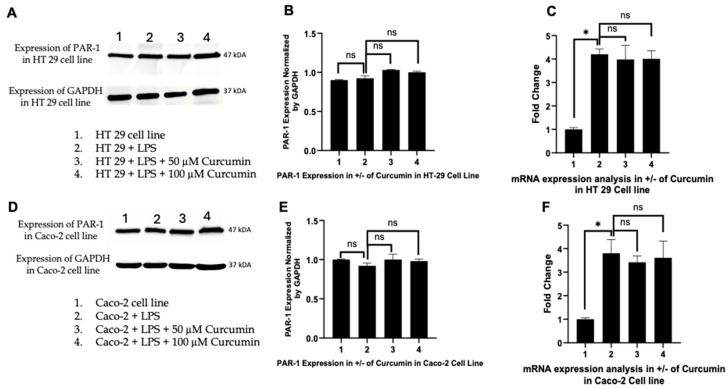
Curcumin does not significantly modulate PAR-1 expression at the protein or mRNA level in HT 29 and Caco-2 cells. This figure examines whether curcumin’s inhibitory effect on PAR-2 is selective, by evaluating the expression of PAR-1, a homologous G-protein-coupled receptor. The rationale for choosing PAR-1 as a control comparator is detailed in the Methods section. (**A**) Representative Western blot analysis showing the expression of PAR-1 protein in HT 29 cells under four conditions: untreated control (Lane 1), LPS-treated (10 µg/mL; Lane 2), LPS + 50 µM curcumin (Lane 3), and LPS + 100 µM curcumin (Lane 4). *GAPDH* was used as a loading control. The molecular weight of the PAR-1 band was observed at ~47 kDa, consistent with expected unglycosylated core protein; (**B**) Densitometric quantification of PAR-1 protein expression in HT 29 cells, normalized to *GAPDH*. No statistically significant differences were observed between groups. (**C**) Quantitative real-time PCR (qPCR) analysis of PAR-1 mRNA expression in HT 29 cells using the 2^−ΔΔCt^ method. Consistent with protein-level findings, mRNA expression remained stable across all treatment conditions; (**D**) Western blot of PAR-1 expression in Caco-2 cells under identical experimental conditions. The protein band for PAR-1 remained unchanged across treatments; (**E**) Densitometric analysis of Caco-2 PAR-1 expression normalized to *GAPDH* shows no significant variation; (**F**) qPCR analysis of PAR-1 mRNA levels in Caco-2 cells further confirms the absence of significant transcriptional modulation following curcumin treatment. (Note: Data in panels B, C, E, and F represent means ± SEM from triplicate experiments (*n* = 3). One-way ANOVA followed by Tukey’s post hoc test was used to assess statistical significance. All comparisons showed non-significant changes (* *p* < 0.05), underscoring the specificity of curcumin’s action on PAR-2 rather than across the broader PAR family, ns: not significant).

In HT 29 cells, Western blot analysis revealed stable PAR-1 expression across all experimental conditions (Figure 3A). Densitometric quantification (Figure 3B) confirmed no statistically significant differences between untreated controls, LPS-stimulated cells, and curcumin-treated groups. The integrity of protein loading was validated by consistent *GAPDH* expression across lanes. Quantitative real-time PCR (qPCR) analysis further corroborated these findings, with PAR-1 mRNA levels exhibiting no significant modulation following curcumin exposure (Figure 3C). These data suggest that, unlike PAR-2, PAR-1 is not transcriptionally or translationally downregulated by curcumin in the HT 29 model.

A similar pattern was observed in Caco-2 cells. Western blot analysis (Figure 3D) showed uniform PAR-1 expression across all groups, including LPS-treated cells and those co-treated with curcumin. Densitometric evaluation normalized to *GAPDH* (Figure 3E) demonstrated no statistically significant changes in protein expression. This stability in PAR-1 expression was further supported by qPCR analysis (Figure 3F), which showed consistent mRNA levels regardless of curcumin treatment.

Together, these results reinforce the specificity of curcumin’s inhibitory action on PAR-2 expression. The unaltered expression of PAR-1 at both the transcript and protein levels in two distinct CRC cell lines under matched inflammatory conditions suggests that curcumin selectively targets the PAR-2 signaling axis without broadly suppressing the PAR family. This finding underscores the potential mechanistic specificity of curcumin in modulating protease-activated receptor–mediated inflammatory signaling in CRC models.

### 3.4. Curcumin Modulates ERK1/2 and Phosphorylated ERK Signaling in HT 29 and Caco-2 Cells

To investigate whether curcumin modulates MAPK pathway signaling downstream of PAR-2 in LPS-induced inflammatory conditions, we assessed the expression of ERK1, ERK2, and their phosphorylated forms (p-ERK1/2) in HT 29 and Caco-2 CRC cells, treated with LPS alone or in combination with curcumin (50 µM or 100 µM). The selection of ERK isoforms was based on their established role in promoting inflammatory cytokine production, cell proliferation, and survival in CRC models downstream of PAR-2 activation [134].

**Figure 4:**
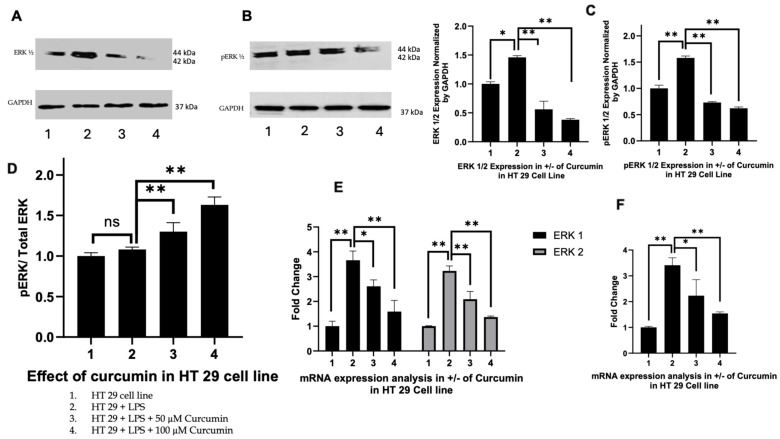
Curcumin attenuates LPS-induced ERK1/2 and phosphorylated ERK (p-ERK) expression in HT 29 colorectal cancer cells. (**A**) Representative Western blot images showing the expression of total ERK1/2 (top left) and phosphorylated ERK1/2 (p-ERK; top right) in HT 29 cells under four conditions: untreated (Lane 1), LPS-treated (10 µg/mL; Lane 2), LPS + 50 µM curcumin (Lane 3), and LPS + 100 µM curcumin (Lane 4). *GAPDH* (37 kDa) served as the internal loading control; (**B**,**C**) Densitometric quantification of total ERK1/2 (**B**) and p-ERK1/2 (**C**) bands normalized to *GAPDH*. Curcumin elicited a dose-dependent suppression of both total and phosphorylated ERK1/2 levels; (**D**) Quantification of the p-ERK to total ERK ratio reveals an apparent increase in phosphorylation status upon curcumin treatment despite suppression of total protein levels, suggesting a relative enrichment of p-ERK; (**E**) Real-time PCR analysis of ERK1 (MAPK3) and ERK2 (MAPK1) mRNA expression shows significant upregulation following LPS treatment and downregulation with curcumin in a concentration-dependent manner; (**F**) Transcriptional modulation of p-ERK was assessed by evaluating DUSP6 mRNA expression, which serves as a proxy for MAPK signaling activity. Curcumin significantly attenuated DUSP6 expression in LPS-primed cells. (Note: Data are presented as mean ± SEM of triplicate experiments. Statistical comparisons were performed using one-way ANOVA followed by Tukey’s post hoc test. *p* < 0.05 (*), *p* < 0.01 (**), ns: not significant).

HT 29 cells: As illustrated in Figure 4A, Western blot analysis revealed that both ERK1 and ERK2 protein levels were significantly upregulated upon LPS stimulation (lane 2) compared to untreated controls (lane 1). Curcumin co-treatment led to a dose-dependent reduction in ERK1/2 expression, with 100 µM curcumin yielding the most pronounced suppression (lanes 3 and 4). Phosphorylated ERK (p-ERK) levels followed a similar trend, being elevated by LPS but attenuated by curcumin, although residual p-ERK signal persisted at higher curcumin doses. Densitometric analysis (Figure 4B,C) quantified these changes, showing a clear reduction in both total ERK1/2 and p-ERK levels. Notably, when analyzed as a ratio of phosphorylated to total ERK (p-ERK:ERK), a divergent trend emerged (Figure 4D), where the ratio increased slightly with curcumin treatment despite a decline in absolute p-ERK levels, suggesting potential post-translational modulation of ERK signaling.

**Figure 5:**
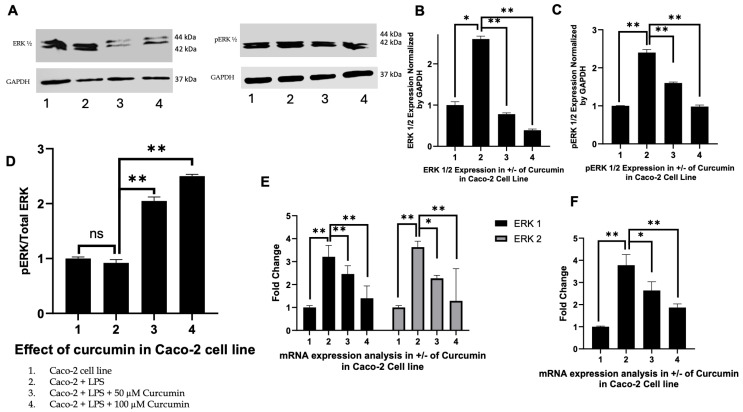
Curcumin attenuates LPS-induced ERK1/2 and phosphorylated ERK (p-ERK) expression in Caco-2 cells. (**A**) Representative Western blot images showing total ERK1/2 and p-ERK protein levels in Caco-2 cells across four treatment conditions: untreated control (Lane 1), LPS-treated (10 µg/mL; Lane 2), LPS + curcumin 50 µM (Lane 3), and LPS + curcumin 100 µM (Lane 4). *GAPDH* was used as the loading control; (**B**,**C**) Densitometric quantification of Western blot bands for total ERK1/2 (**B**) and p-ERK (**C**), normalized to *GAPDH*; (**D**) Ratio of phosphorylated ERK to total ERK was computed for each condition to assess relative pathway activation; (**E**) Relative mRNA expression levels of ERK1 and ERK2 in the same treatment groups, as measured by qPCR and normalized to *GAPDH*; (**F**) Transcript levels of DUSP6, a downstream ERK-responsive phosphatase, were quantified by qPCR. Data are expressed as fold change relative to untreated controls using the 2^−ΔΔCt^ method. (Note: All experiments were performed in biological triplicates (*n* = 3). Statistical significance was assessed by one-way ANOVA with Tukey’s post hoc test; * *p* < 0.05; ** *p* < 0.01, ns: not significant).

Caco-2 cells: A similar pattern was observed in Caco-2 cells (Figure 5A). LPS exposure (lane 2) led to pronounced upregulation of total ERK1/2, which was attenuated by curcumin in a dose-dependent manner at 50 µM and 100 µM (lanes 3 and 4). Quantitative densitometry (Figure 5B) confirmed a statistically significant reduction in total ERK1/2 levels upon curcumin treatment (*p* < 0.01), with a stronger effect at 100 µM. The expression of p-ERK (Figure 5A) similarly increased with LPS but was reduced by curcumin (Figure 5C), with no enrichment in the p-ERK:ERK ratio (Figure 5D), indicating proportional downregulation of both total and active ERK isoforms.

Transcriptional effects: In both HT 29 and Caco-2 cells, RTPCR analysis of ERK1 (MAPK3) and ERK2 (MAPK1) mRNA (Figure 4E and Figure 5E) mirrored the protein-level observations. LPS stimulation upregulated ERK1/2 transcripts, whereas curcumin co-treatment significantly suppressed their expression in a dose-dependent manner. ERK1 was more responsive to curcumin compared to ERK2, particularly in Caco-2 cells. Furthermore, DUSP6, a dual-specificity phosphatase and downstream target of MAPK signaling—was significantly downregulated following curcumin treatment (Figure 4F and Figure 5F), aligning with the observed suppression of ERK activation at the protein level.

Therefore, taken together, these findings demonstrate that curcumin effectively suppresses both total and phosphorylated ERK signaling components in LPS-induced HT 29 and Caco-2 cells, with consistent effects observed at both transcriptional and post-translational levels. The subtle shift in the p-ERK:ERK ratio in HT 29 cells suggests a complex feedback mechanism that may be partially disrupted by curcumin. These results underscore the conserved anti-inflammatory and anti-proliferative potential of curcumin via modulation of MAPK signaling in CRC models.

### 3.5. Curcumin Attenuates TNF-α Expression in LPS-Stimulated HT 29 and Caco-2 Cells

Following the observation that curcumin attenuates PAR-2 and ERK1/2 signaling, we next investigated TNF-α as it represents a critical downstream effector that integrates pro-inflammatory signaling with apoptotic regulation [135]. TNF-α is transcriptionally regulated by MAPK and NF-κB pathways, both of which are activated by PAR-2 and ERK1/2, and serves as a proximal trigger for *caspase-8* activation and mitochondrial apoptotic signaling [136,137,138]. Placing TNF-α analysis after ERK evaluation allows a mechanistic transition from upstream receptor-kinase signaling to effector cytokine responses, which are ultimately linked to the apoptotic outcomes assessed later.

**Figure 6:**
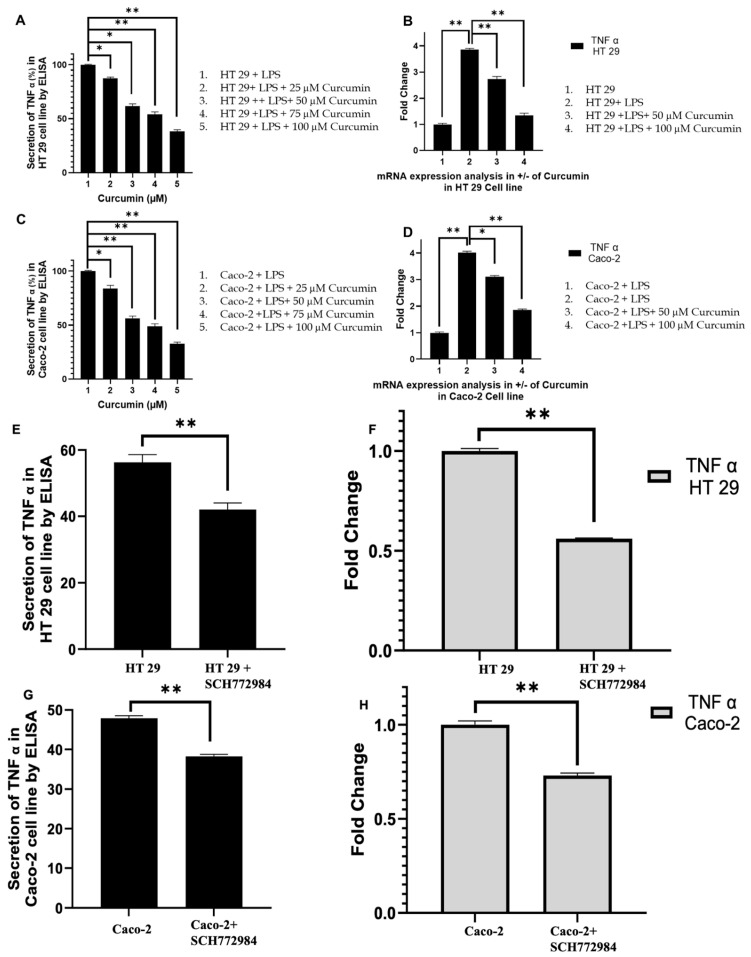
Curcumin attenuates TNF-α expression in LPS-stimulated HT 29 and Caco-2 cells. (**A**) ELISA quantification of TNF-α secretion in HT 29 cells demonstrates significant LPS-induced upregulation, which is dose-dependently reduced by curcumin treatment (25, 50, 75, and 100 μM), with maximal suppression at 100 μM (** *p* < 0.01); (**B**) RT-PCR analysis in HT 29 cells reveals that LPS robustly induces TNF-α mRNA expression, which is significantly downregulated by curcumin in a concentration-dependent manner (** *p* < 0.01); (**C**) In Caco-2 cells, ELISA reveals a similar LPS-induced increase in TNF-α secretion that is significantly attenuated by curcumin, with the most potent suppression observed at 100 μM (** *p* < 0.01); (**D**) RT-PCR data in Caco-2 cells show that curcumin significantly inhibits LPS-induced TNF-α mRNA expression across all tested concentrations, with the greatest inhibition at 100 μM (** *p* < 0.01); (**E**) Treatment of HT 29 cells with the selective ERK inhibitor SCH772984 (1 μM) markedly reduces TNF-α secretion following LPS stimulation, as measured by ELISA (** *p* < 0.01); (**F**) RT-PCR analysis in HT 29 cells demonstrates that SCH772984 also significantly downregulates TNF-α mRNA levels in the presence of LPS (** *p* < 0.01); (**G**) In Caco-2 cells, SCH772984 treatment similarly suppresses LPS-induced TNF-α protein secretion (** *p* < 0.01); (**H**) RT-PCR data confirm that SCH772984 significantly reduces TNF-α mRNA expression in LPS-stimulated Caco-2 cells (** *p* < 0.01). (Note: Data are expressed as mean ± SEM of three independent experiments; significance was assessed via one-way ANOVA followed by Tukey’s post hoc test. In all the experiments * *p* < 0.05).

Curcumin markedly suppressed TNF-α at both protein and mRNA levels in LPS-stimulated HT 29 and Caco-2 cells in a dose-dependent manner. In HT 29 cells, LPS induced a significant increase in TNF-α secretion (Figure 6A), which was progressively reduced by curcumin, with maximal inhibition observed at 100 μM (*p* < 0.01). RT-PCR analysis revealed a parallel trend in TNF-α transcripts (Figure 6B). Similarly, in Caco-2 cells, curcumin treatment significantly reduced both TNF-α secretion (Figure 6C) and mRNA expression (Figure 6D) compared to the LPS group, confirming its dual transcriptional and post-transcriptional regulation of TNF-α. To establish whether this suppression is mediated through ERK signaling, we employed the selective ERK inhibitor SCH772984. ELISA and RT-PCR analyses demonstrated that SCH772984 markedly decreased TNF-α protein (Figure 6E,G) and mRNA levels (Figure 6F,H) in both HT 29 and Caco-2 cells, reinforcing the mechanistic link between ERK inhibition and TNF-α downregulation. These findings underscore that curcumin’s anti-inflammatory action is at least partly mediated through suppression of the PAR-2/ERK/TNF-α axis, bridging upstream kinase signaling to downstream apoptotic pathways (refer below).

### 3.6. Curcumin Induces Apoptosis in HT 29 and Caco-2 Cells via PAR-2/ERK/TNF-α-Mediated Activation of Extrinsic and Intrinsic Pathways

Having established that curcumin suppresses PAR-2 and ERK1/2 signaling and downregulates TNF-α expression, we next examined whether these upstream alterations culminate in activation of apoptotic pathways. PAR-2 signaling is well-documented to confer apoptosis resistance by engaging MEK1/2–ERK1/2 and PI3K–Akt cascades, which suppress extrinsic and intrinsic apoptotic mechanisms through inhibition of initiator caspases (e.g., *caspase-8*), modulation of the *Bcl 2* family, and prevention of mitochondrial outer membrane permeabilization (MOMP) [139]. Therefore, targeting PAR-2 provides a mechanistic entry point for reactivating apoptosis, and we hypothesized that curcumin’s suppression of the PAR-2–ERK axis would reverse these anti-apoptotic effects.

**Figure 7:**
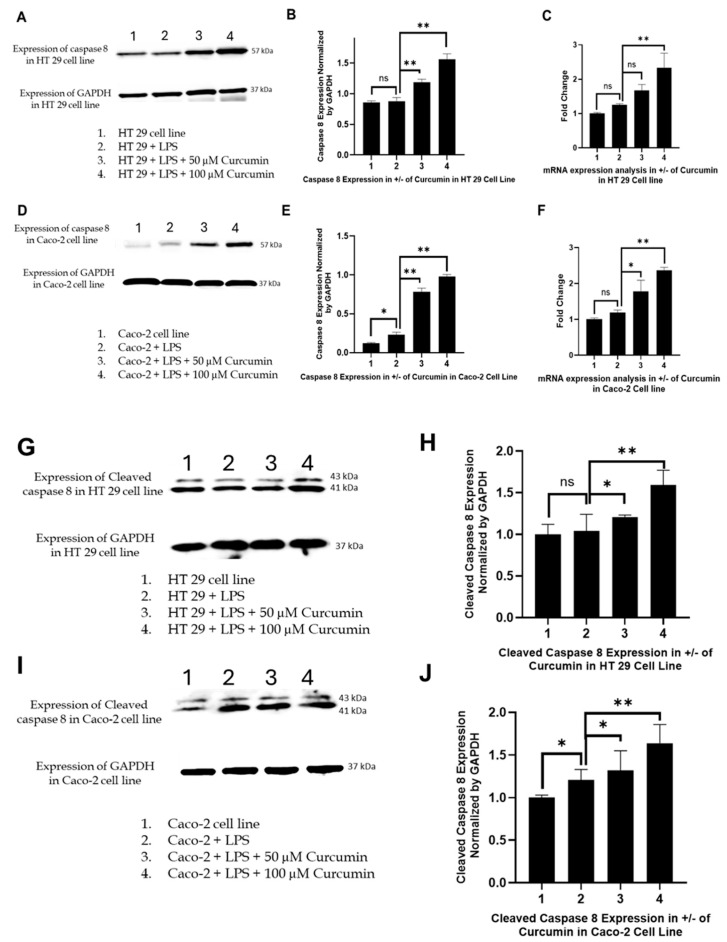
Curcumin restores *caspase-8* expression suppressed by LPS-induced PAR-2 activation in colorectal cancer cell lines. HT 29 (upper panel; **A**–**C**) and Caco-2 (lower panel; **D**–**F**) cells were exposed to LPS (10 µg/mL) to simulate inflammatory conditions and subsequently treated with curcumin (50 µM and 100 µM) to assess effects on *caspase-8* expression. (**A**,**D**) Representative Western blot images showing full-length *caspase-8* protein levels. *GAPDH* was used as a loading control. (**B**,**E**) Densitometric analysis of *caspase-8* bands normalized to *GAPDH*, expressed as fold change relative to untreated controls. (**C**,**F**) Relative mRNA expression levels of CASP8 determined by real-time PCR, normalized to *GAPDH*, confirming transcriptional upregulation by curcumin. (**G**,**I**) Western blot detection of cleaved (activated) *caspase-8* fragments under the same conditions, demonstrating that curcumin induces *caspase-8* activation in a dose-dependent manner. (**H**,**J**) Quantification of cleaved *caspase-8* bands normalized to *GAPDH*, confirming that curcumin relieves the LPS-mediated blockade of *caspase-8* activation, particularly at 100 µM.(Note: In both cell lines, LPS reduced *caspase-8* expression, while curcumin treatment markedly reversed this suppression in a concentration-dependent manner. All data are expressed as mean ± SEM from three independent experiments. Statistical analysis was performed using one-way ANOVA with Tukey’s post hoc test. ns: not significant, * *p* < 0.05 and ** *p* < 0.01, versus LPS-treated controls.

Curcumin restores *caspase-8* activation: *Caspase-8*, the apical initiator of the extrinsic apoptotic pathway, is tightly suppressed by PAR-2 signaling through phosphorylation of pro-apoptotic proteins (e.g., *BAD*) and the induction of anti-apoptotic molecules such as MCL-1 and *Bcl2L12* [140]. To differentiate between basal expression and proteolytic activation, both full-length and cleaved caspase-8 were examined. Western blot analysis (Figure 7A,D,G,I) showed that LPS treatment markedly reduced both forms of caspase-8 in HT-29 and Caco-2 cells, reflecting PAR-2-mediated inhibition of extrinsic apoptosis. In contrast, curcumin treatment (50 and 100 µM) significantly restored full-length caspase-8 expression and, importantly, induced its cleavage, the hallmark of enzymatic activation (Figure 7A,D,G,I). Although canonical apoptosis is typically associated with depletion of the procaspase pool and accumulation of cleaved fragments, in our system the increase in both full-length and cleaved caspase-8 suggests that curcumin not only triggers activation but also transcriptionally up-regulates *CASP8*, thereby replenishing substrate while simultaneously promoting its cleavage. This interpretation is supported by our parallel RT-PCR results (Figure 7C,F), which demonstrated a dose-dependent rise in CASP8 transcripts. Thus, curcumin appears to act at both transcriptional and post-translational levels to relieve PAR-2/ERK-mediated suppression of the extrinsic death receptor pathway, reinstating caspase-8 activity and apoptotic priming.

**Figure 8:**
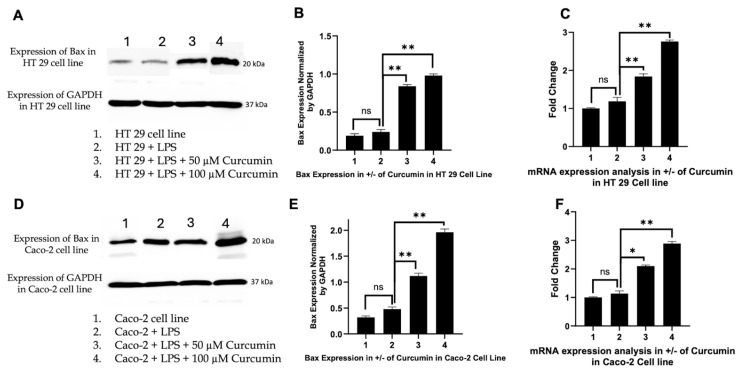
Curcumin induces *Bax* expression to activate the intrinsic apoptotic pathway in LPS-stimulated CRC cell lines. Western blot and RT-PCR analyses were performed to assess the effect of curcumin on *Bax* expression in HT 29 and Caco-2 cells pre-treated with LPS. (**A**) Representative Western blot showing *Bax* protein levels in HT 29 cells across untreated, LPS-only, and curcumin-treated (50 µM and 100 µM) conditions. *GAPDH* was used as a loading control; (**B**) Densitometric analysis of *Bax* expression normalized to *GAPDH* in HT 29 cells; (**C**) mRNA expression of *Bax* in HT 29 cells assessed by RT-PCR, demonstrating transcriptional upregulation by curcumin; (**D**) Representative Western blot for *Bax* expression in Caco-2 cells under the same treatment conditions; (**E**) Densitometric quantification in Caco-2 confirms a dose-dependent increase in *Bax* protein levels with curcumin; (**F**) RT-PCR analysis of *Bax* mRNA in Caco-2 cells, indicating transcriptional upregulation in response to curcumin. (Note: Data represent the mean ± SD of three independent experiments. * *p* < 0.05 and ** *p* < 0.01, ns: not significant).

**Figure 9:**
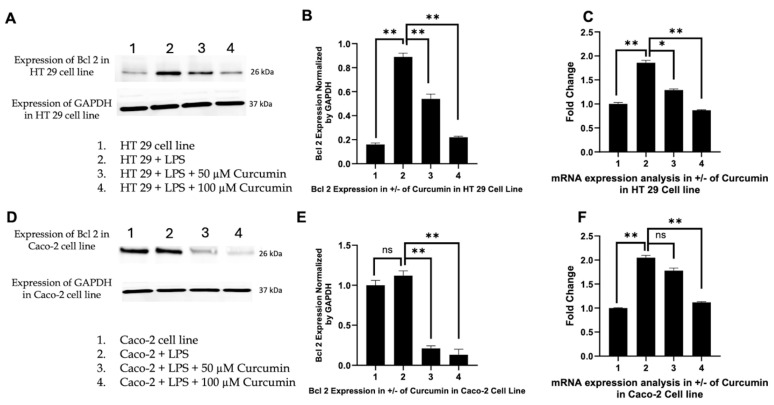
Curcumin Downregulates *Bcl 2* Expression in LPS-Stimulated HT 29 and Caco-2 Cells. (**A**) Western blot showing *Bcl 2* protein expression in HT 29 cells under control, LPS, LPS + curcumin 50 µM, and LPS + curcumin 100 µM conditions; (**B**) Densitometric quantification of *Bcl 2* protein levels in HT 29 normalized to *GAPDH*, confirming dose-dependent suppression by curcumin; (**C**) RT-PCR analysis of *Bcl 2* mRNA in HT 29 showing LPS-induced upregulation reversed by curcumin; (**D**) Western blot of *Bcl 2* protein expression in Caco-2 cells under identical treatment conditions demonstrating curcumin-mediated suppression; (**E**) Densitometric analysis of *Bcl 2* protein in Caco-2 cells normalized to *GAPDH* showing significant reduction with curcumin; (**F**) RT-PCR analysis of *Bcl 2* mRNA in Caco-2 cells reflecting the protein data with transcript downregulation upon curcumin treatment. (Note: Data represent the mean ± SD of three independent experiments. * *p* < 0.05 and ** *p* < 0.01, ns: not significant).

Induction of *Bax* and downregulation of *Bcl 2*: Activated *caspase-8* cleaves Bid, linking the extrinsic pathway to mitochondrial apoptosis by inducing *Bax* oligomerization and MOMP [141]. We therefore investigated *Bax* expression to determine whether curcumin-mediated *caspase-8* activation translated into intrinsic pathway engagement. In HT 29 and Caco-2 cells, Western blotting revealed negligible *Bax* induction with LPS alone, whereas curcumin co-treatment, especially at 100 µM, produced a pronounced increase in *Bax* protein levels (Figure 8A,D), confirmed by densitometric analysis (Figure 8B,E). RTPCR further showed significant transcriptional upregulation of *Bax* (Figure 8C,F), indicating curcumin-mediated activation at both levels. In parallel, we assessed *Bcl 2*, the primary anti-apoptotic counterpart of *Bax*. LPS stimulation markedly upregulated *Bcl 2* expression (Figure 9A,D), consistent with a pro-survival phenotype. Curcumin treatment significantly and dose-dependently reduced *Bcl 2* protein and mRNA levels (Figure 9B,C,E,F), effectively shifting the *Bax*/*Bcl 2* ratio in favor of apoptosis. These data highlight a concerted effect of curcumin on both arms of the mitochondrial checkpoint, tilting the balance toward MOMP and cytochrome c release.

**Figure 10:**
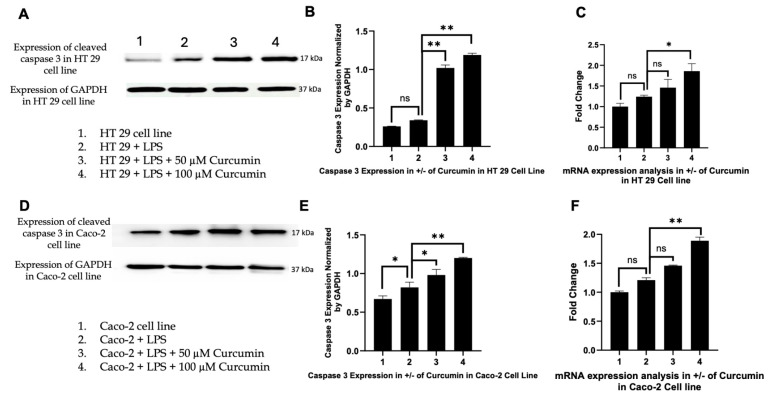
Curcumin upregulates *caspase-3* expression, promoting execution-phase apoptosis in inflamed CRC cell line. Western blot and RT-PCR were used to evaluate the effect of curcumin on *caspase-3* expression in LPS-stimulated HT 29 and Caco-2 cells. (**A**) Western blot showing dose-dependent increase in *caspase-3* protein expression in HT 29 cells after treatment with 50 µM and 100 µM curcumin; (**B**) Densitometric analysis of *caspase-3* expression normalized to *GAPDH* in HT 29 cells; (**C**) CASP3 mRNA expression levels in HT 29 cells, as determined by real-time PCR, confirming transcriptional upregulation with curcumin; (**D**) Western blot analysis of *caspase-3* expression in Caco-2 cells under similar treatment conditions; (**E**) Densitometric quantification of Caco-2 *caspase-3* protein normalized to *GAPDH*; (**F**) RT-PCR analysis of CASP3 mRNA levels in Caco-2 cells showing no significant change with LPS alone but robust upregulation with curcumin. (Note: Data represent the mean ± SD of three independent experiments. * *p* < 0.05 and ** *p* < 0.01, ns: not significant).

Activation of *caspase-3* as the executioner caspase: Given that *Bax*-mediated MOMP triggers apoptosome assembly and subsequent *caspase-3* activation [142], we next analyzed *caspase-3* expression. Curcumin induced a robust, concentration-dependent increase in *caspase-3* protein in both HT 29 and Caco-2 cells, with 100 µM curcumin producing the strongest upregulation (Figure 10A,D). Densitometric analyses (Figure 10B,E) confirmed these trends. While LPS alone elicited only a minor, non-significant increase in *caspase-3* protein, no corresponding rise in CASP3 mRNA was detected, suggesting post-transcriptional regulation. In contrast, curcumin treatment significantly upregulated CASP3 mRNA (Figure 10C,F), indicating that the executioner phase of apoptosis is transcriptionally reactivated by curcumin in an inflammatory context.

**Figure 11:**
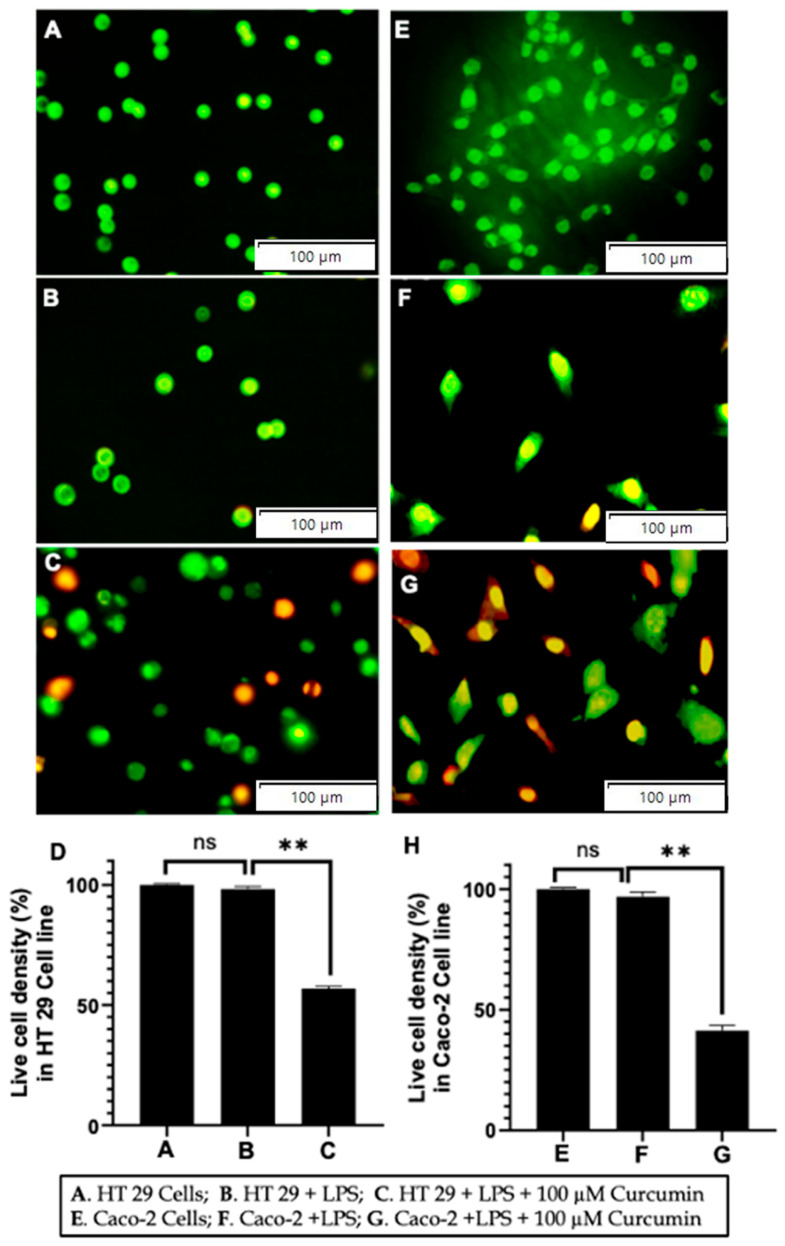
Acridine Orange/Ethidium Bromide Staining Reveals Curcumin-Induced Apoptosis in LPS-Stimulated HT 29 and Caco-2 Cells. Representative fluorescence microscopy images of acridine orange/ethidium bromide (AO/EB) dual-stained HT 29 cells: untreated control (**A**); LPS-treated (**B**); LPS + 100 μM curcumin (**C**); and corresponding live cell density quantification (**D**); Representative fluorescence microscopy images of AO/EB-stained Caco-2 cells: untreated control (**E**); LPS-treated (**F**); LPS + 100 μM curcumin (**G**); and corresponding live cell density quantification (**H**); (Note: Green fluorescence indicates viable cells with intact membranes; orange/red fluorescence denotes apoptotic or non-viable cells with compromised membrane integrity; Quantitative data (**D**,**H**) are presented as mean ± SD, with statistical significance determined by ANOVA followed by post hoc testing (** *p* < 0.01); AO/EB staining confirms curcumin-induced apoptosis in both cell lines, supporting molecular findings from *caspase-8*, *Bax*, *Bcl 2*, and *caspase-3* assays, ns: not significant).

Functional validation of apoptosis: To corroborate the molecular evidence of apoptosis, we employed morphological and quantitative assays. Acridine Orange/Ethidium Bromide (AO/EB) staining revealed that untreated and LPS-treated HT 29 and Caco-2 cells displayed predominantly green fluorescence (viable cells), with only rare early apoptotic events (Figure 11A,B,E,F). In contrast, co-treatment with 100 µM curcumin and LPS resulted in widespread orange-to-red fluorescence, indicative of apoptotic or necrotic nuclei (Figure 11C,G). Quantification of live cell density (Figure 11D,H) demonstrated a significant reduction in viable cells (*p* < 0.01) in curcumin-treated groups compared to both control and LPS-only conditions.

**Figure 12:**
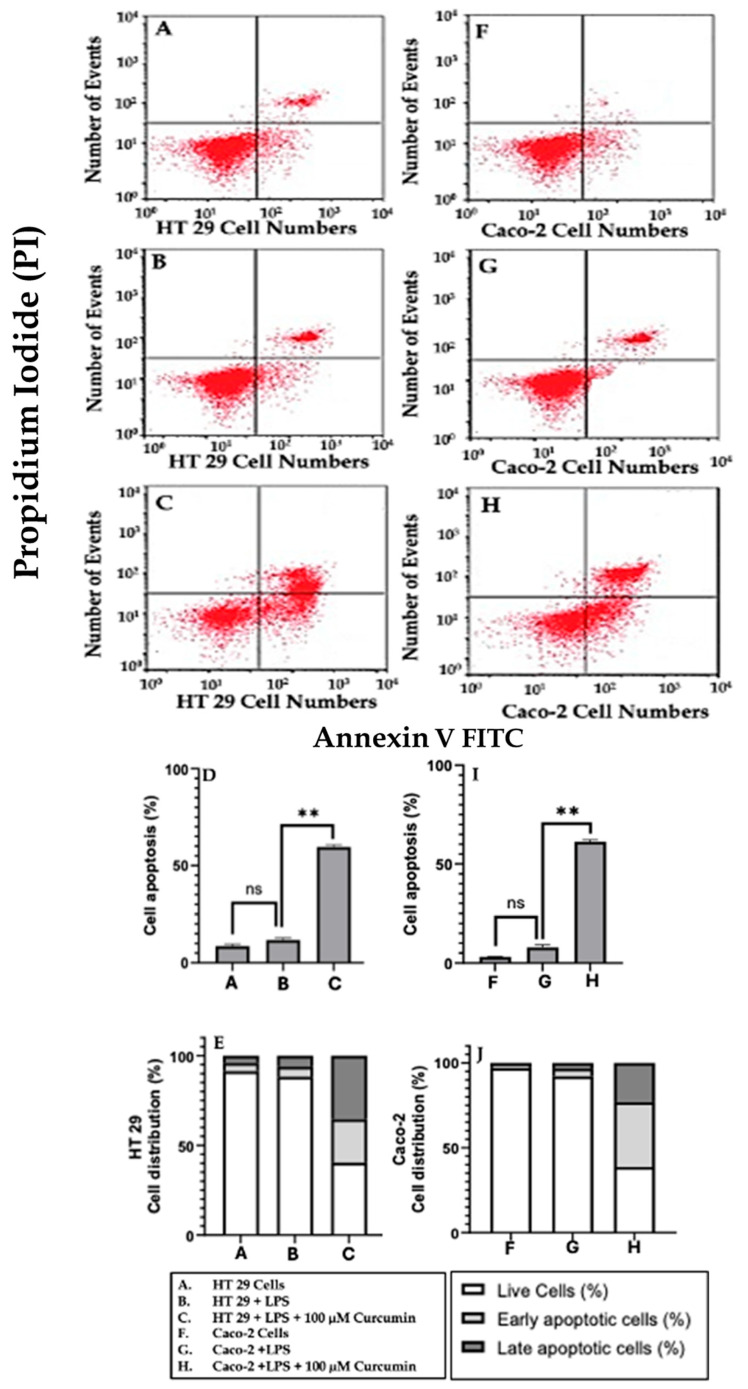
Curcumin Induces Quantitative Apoptosis in LPS-Stimulated HT 29 and Caco-2 Cells as Assessed by Annexin V/PI Flow Cytometry. Representative flow cytometry plots showing Annexin V-FITC and PI staining in HT 29 cells under untreated (**A**), LPS-treated (**B**), and LPS + 100 μM curcumin (**C**) conditions; Quantitative analysis of total apoptotic cells in HT 29 populations under each treatment (**D**); Cell distribution analysis showing percentages of viable, early apoptotic, late apoptotic, and necrotic cells in HT 29 (**E**); Representative flow cytometry plots for Caco-2 cells under untreated (**F**), LPS-treated (**G**), and LPS + 100 μM curcumin (**H**) conditions; Quantitative apoptotic cell percentages in Caco-2 populations (**I**); Corresponding distribution across viability and apoptosis states in Caco-2 (**J**); (Note: Annexin V-positive, PI-negative cells represent early apoptosis; Annexin V and PI double-positive cells indicate late apoptosis; Data are presented as mean ± SD from independent experiments; ** *p* < 0.01 vs. untreated or LPS-treated groups; ns = not significant). Results confirm that curcumin treatment significantly increases both early and late apoptosis in LPS-stimulated HT 29 and Caco-2 cells.

To further quantify apoptosis, Annexin V/PI flow cytometry was performed. In both cell lines, curcumin treatment led to a marked increase in early apoptotic (Annexin V+/PI–) and late apoptotic (Annexin V+/PI+) populations compared to LPS or untreated groups (Figure 12A–C,F–H). Quantitative analysis (Figure 12D,E,I,J) revealed a statistically significant (*p* < 0.01) increase in total apoptotic cells, confirming that curcumin robustly drives apoptotic progression.

**Figure 13:**
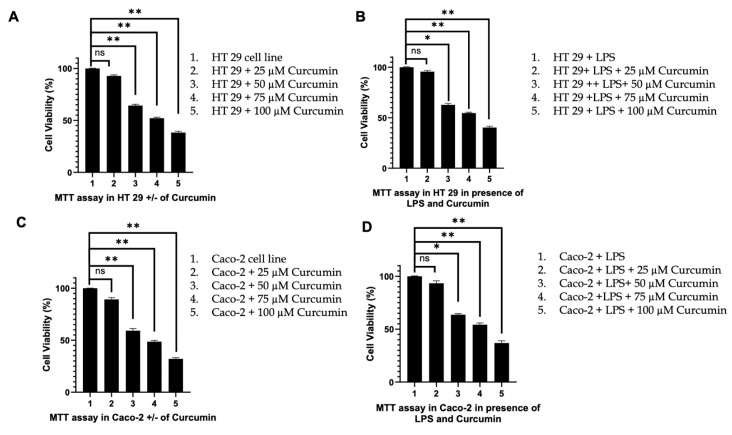
Curcumin reduces cell viability in HT 29 and Caco-2 cells under basal and LPS-stimulated conditions as measured by MTT assay. (**A**) Dose-dependent reduction in cell viability in HT 29 cells following curcumin treatment (25, 50, 75, 100 µM) under basal conditions, as measured by MTT absorbance at 570 nm; (**B**) Curcumin significantly reduces HT 29 cell viability in the presence of LPS (10 µg/mL), with maximal cytotoxicity at 100 µM; (**C**) Caco-2 cells show a comparable dose-dependent decline in viability under basal conditions, with statistically significant reductions at all tested concentrations of curcumin; (**D**) Under LPS-stimulated conditions, curcumin markedly decreases viability in Caco-2 cells, indicating robust cytotoxicity in an inflammatory microenvironment. (Note: All values are expressed as mean ± SEM from three independent experiments; significance determined using one-way ANOVA with Tukey’s post hoc test (ns: non-significant, * *p* < 0.05; ** *p* < 0.01)).

Reduction in cell viability (MTT assay): Finally, to integrate the molecular and functional outcomes into a global measure of cytotoxicity, we conducted MTT assays in the presence and absence of LPS. Curcumin treatment resulted in a dose-dependent reduction in mitochondrial metabolic activity, with maximal cytotoxicity observed at 100 µM in both HT 29 and Caco-2 cells (Figure 13A–D). LPS alone had minimal effect on viability, but its combination with curcumin further exacerbated cell death, suggesting that curcumin’s pro-apoptotic activity overrides LPS-driven survival cues.

In summary, these findings establish a coherent mechanistic sequence in which curcumin suppresses PAR-2 and ERK/TNF-α signaling, thereby reactivating both extrinsic (*caspase-8*) and intrinsic (*Bax*/*Bcl 2*) apoptotic pathways. The convergence of these pathways leads to *caspase-3* activation, culminating in apoptotic cell death as validated by AO/EB staining, Annexin V/PI flow cytometry, and MTT assays. This multi-level regulation underscores curcumin’s potential as a pleiotropic anti-inflammatory and pro-apoptotic agent in colorectal cancer models.

### 3.7. Effect of Curcumin on Calcium Dynamics

After demonstrating that curcumin downregulates PAR-2 and TNF-α, modulates caspase activation, and shifts the balance between pro-and anti-apoptotic proteins (*Bax* and *Bcl 2*), we next assessed intracellular calcium dynamics using the Fluo-4 assay in HT 29 and Caco-2 cells. This transition was guided by the well-established mechanistic link between PAR-2 activation and intracellular calcium release via the PLC/InsP_3_ pathway, which not only amplifies inflammatory signaling through NF-κB but also directly influences mitochondrial apoptotic checkpoints such as *Bax*/*Bcl 2* and *caspase-3* activation [122]. Calcium is a central second messenger in both inflammatory and apoptotic signaling, with PAR-2 and TNF-α known to stimulate intracellular calcium release and influx. Dysregulation of calcium homeostasis can trigger mitochondrial dysfunction, amplify apoptotic signaling, and contribute to cellular injury. By examining changes in Fluo-4 fluorescence, we aimed to determine whether curcumin’s anti-inflammatory and pro-apoptotic actions in these intestinal epithelial cell lines are associated with modulation of calcium signaling pathways, thereby providing further mechanistic insight into its cellular effects.

**Figure 14:**
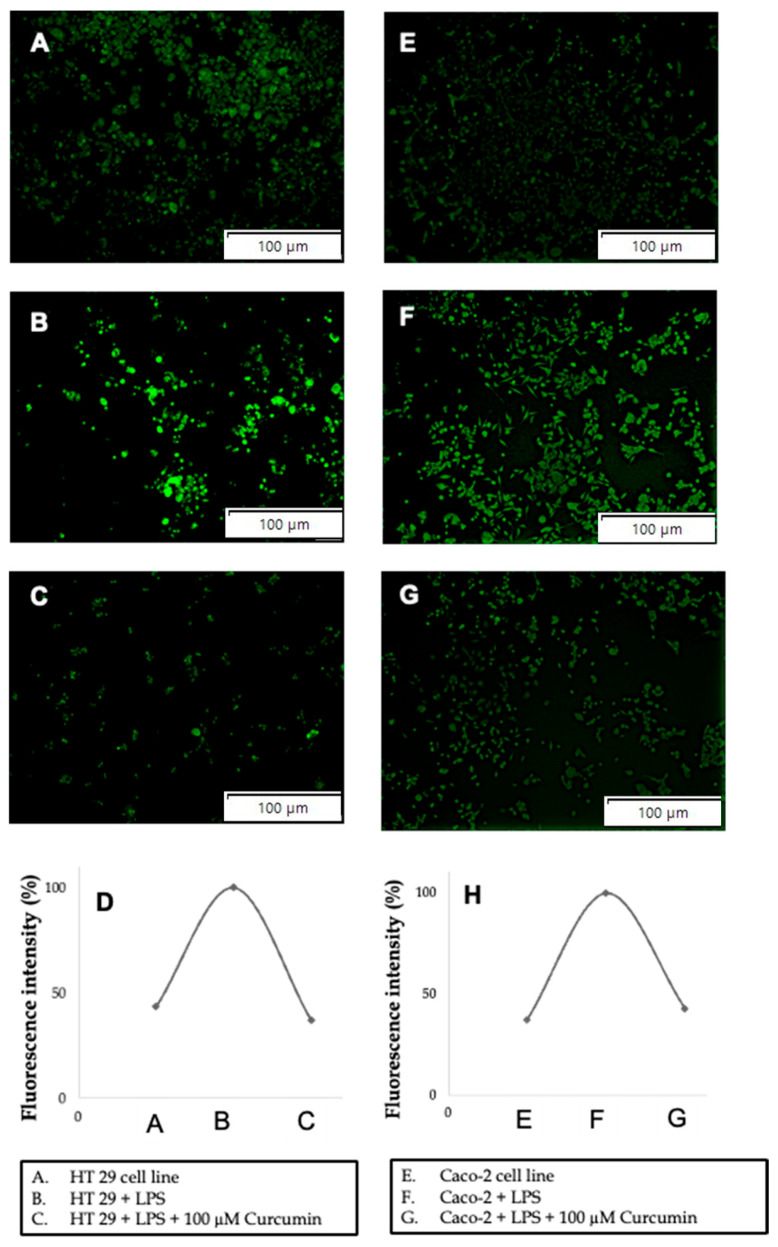
Curcumin attenuates LPS-induced intracellular calcium elevation in HT 29 and Caco-2 cells as assessed by Fluo-4 fluorescence imaging. (**A**) Baseline Fluo-4 fluorescence in untreated HT 29 cells reveals moderate intracellular calcium levels; (**B**) LPS stimulation (10 µg/mL) leads to a marked increase in intracellular calcium as indicated by elevated Fluo-4 fluorescence; (**C**) Co-treatment with 100 µM curcumin significantly reduces LPS-induced calcium elevation in HT 29 cells; (**D**) Quantitative fluorescence analysis confirms statistically significant suppression of LPS-induced calcium increase by curcumin in HT 29 cells); (**E**) Untreated Caco-2 cells exhibit modest baseline Fluo-4 fluorescence; (**F**) LPS treatment induces substantial elevation of intracellular calcium in Caco-2 cells; (**G**) Curcumin (100 µM) markedly diminishes calcium levels in LPS-treated Caco-2 cells; (**H**) Quantitative analysis in Caco-2 confirms significant curcumin-mediated attenuation of calcium flux. (Note: Data represent mean ± SEM of three independent experiments; statistical significance was determined using one-way ANOVA followed by Tukey’s post hoc test).

Fluo-4 fluorescence imaging revealed that untreated HT 29 cells (Figure 14A) displayed moderate baseline fluorescence, corresponding to physiological intracellular calcium levels. Upon LPS stimulation (Figure 14B), there was a marked increase in Fluo-4 fluorescence intensity, indicating a substantial elevation in intracellular calcium. Notably, treatment with 100 μM curcumin in the presence of LPS (Figure 14C) resulted in a pronounced reduction in fluorescence intensity, approaching or falling below baseline levels observed in untreated cells. Quantitative analysis (Figure 14D) confirmed that LPS significantly increased intracellular calcium compared to control, while curcumin treatment robustly suppressed this LPS-induced calcium elevation (*p* < 0.01).

Morphologically, HT 29 cells maintained their typical epithelial appearance across all conditions, though LPS stimulation appeared to increase cell clustering and density. Curcumin treatment did not induce overt morphological changes but was associated with a reduction in overall fluorescence, consistent with lower intracellular calcium.

A similar pattern was observed in Caco-2 cells. Baseline Fluo-4 fluorescence in untreated cells (Figure 14E) was modest, while LPS stimulation (Figure 14F) led to a substantial increase in fluorescence intensity, reflecting elevated intracellular calcium. Curcumin treatment (Figure 14G) significantly diminished Fluo-4 fluorescence, restoring calcium levels toward or below those of untreated controls. Quantitative analysis (Figure 14H) mirrored the findings in HT 29 cells, with curcumin significantly reducing LPS-induced calcium elevation (*p* < 0.01).

Caco-2 cells retained their characteristic cobblestone morphology throughout the experiment. LPS exposure did not cause major morphological alterations, but curcumin treatment was associated with a visible decrease in fluorescence intensity, without apparent cytotoxic morphological effects.

In summary, these results demonstrate that curcumin effectively suppresses LPS-induced intracellular calcium elevation in both HT 29 and Caco-2 cells, as evidenced by reduced Fluo-4 fluorescence intensity. This reduction in calcium signaling may contribute to curcumin’s anti-inflammatory and pro-apoptotic effects, further supporting its role in modulating key cellular pathways in intestinal epithelial cells.

### 3.8. Structural Prediction of PAR-2 Using AlphaFold Confirms Canonical GPCR Topology and Identifies a Viable Ligand-Binding Pocket

**Figure 15:**
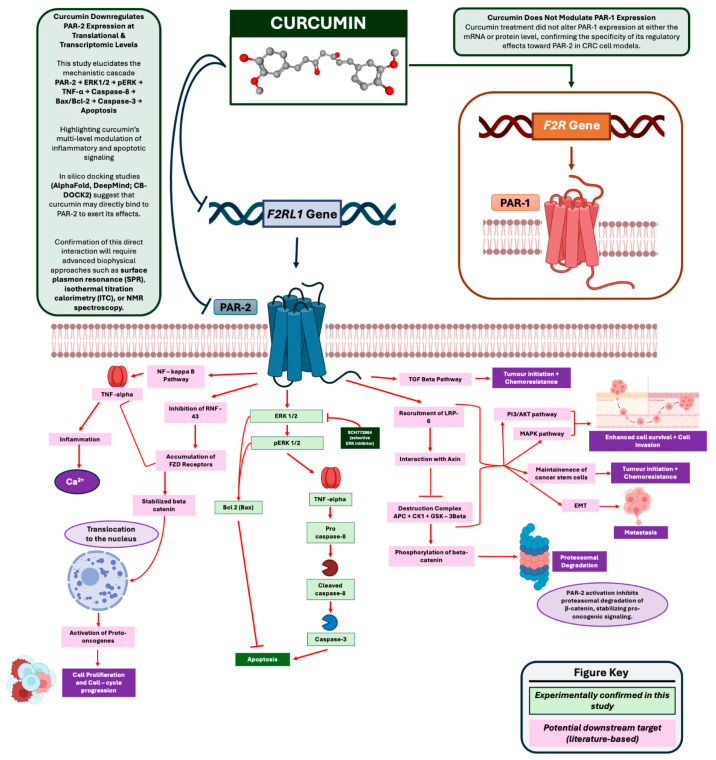
Integrated mechanistic model illustrating the multi-level inhibitory effects of curcumin on PAR-2 signaling and downstream inflammatory and apoptotic pathways in colorectal cancer (CRC) cells. Curcumin is predicted to bind and inhibit *F2RL1* (gene) and its protein product PAR-2, as supported by in silico modeling using CB-Dock2 and DeepMind (AlphaFold2) predictions (upper left). Experimental validation via ITC and SPR is indicated as a future requirement. In contrast, no downregulation of PAR-1 (*F2R*) was observed at either the transcriptional or translational level (upper right). Curcumin-induced inhibition of PAR-2 leads to suppression of pro-inflammatory signaling cascades, including NF-κB activation, TNF-α secretion, and intracellular Ca^2+^ flux, thereby reducing proto-oncogene activation and cell proliferation. The inhibition of MAPK/ERK1/2 signaling (both total and phosphorylated ERK) downstream of PAR-2 results in reduced DUSP6 expression and altered phosphorylation dynamics. This blockade relieves the PAR-2–mediated suppression of apoptosis, enabling activation of *caspase-8* (full-length and cleaved forms), *Bax* upregulation, *Bcl 2* downregulation, and *caspase-3* activation, culminating in apoptosis. Additionally, curcumin disrupts PI3K/Akt and Wnt/β-catenin pathways by inhibiting LRP6 recruitment, β-catenin stabilization, and EMT-related signaling, which collectively impair tumor initiation, chemoresistance, and metastasis. Pathways confirmed experimentally in this study are highlighted with black arrows and red connectors, whereas potential downstream targets derived from literature-based evidence are shown in purple connectors.

Having established through our experimental data that curcumin downregulates PAR-2 and attenuates its downstream signaling (PAR-2 → ERK1/2 → TNF-α → apoptosis), we next sought to investigate whether these effects may also arise from a direct interaction of curcumin with the receptor’s structural framework, thereby altering its conformational dynamics and signaling output. Curcumin, as a polyphenolic diarylheptanoid compound, exhibits a planar and highly conjugated structure with flexible hydroxyl and methoxy groups capable of forming stable hydrophobic interactions, π–π stacking, and hydrogen bonding within transmembrane receptor pockets [143]. This structural versatility enables curcumin to potentially engage PAR-2’s ligand-binding cavity, stabilizing an inactive conformation that prevents the receptor’s conformational cycling required for G-protein coupling and β-arrestin recruitment [144]. Attenuation of PAR-2’s conformational plasticity would disrupt its ability to trigger canonical phosphorylation cascades, thereby suppressing downstream MAPK and NF-κB pathways [145]. NF-κB, a transcription factor central to inflammatory responses, is activated by PAR-2 signaling through TLR4-like cross-talk and ERK1/2 phosphorylation, resulting in nuclear translocation and transcriptional upregulation of pro-inflammatory and pro-survival genes such as TNF-α, *Bcl 2*, and MCL-1 [146]. Thus, if curcumin directly interferes with PAR-2’s conformational state, this would not only inhibit receptor signaling at the plasma membrane but also attenuate transcriptional programs that sustain inflammation, tumor proliferation, and apoptosis resistance. As illustrated in Figure 15, such conformational inhibition could theoretically dampen multiple interconnected pathways, including ERK1/2 phosphorylation, NF-κB nuclear activity, and the downstream regulation of *caspase-8* and mitochondrial apoptotic checkpoints.

This rationale provides the foundation for integrating in silico modeling with our experimental findings, as structural docking analyses can reveal whether curcumin is capable of engaging key transmembrane or extracellular domains of PAR-2, thereby offering a plausible molecular explanation for the downregulation of signaling cascades observed in our CRC cell models. To explore this hypothesis, we generated a full-length structural model of human PAR-2 using AlphaFold2 and subsequently performed molecular docking studies with curcumin to identify energetically favorable binding pockets and assess their potential mechanistic relevance.

**Figure 16:**
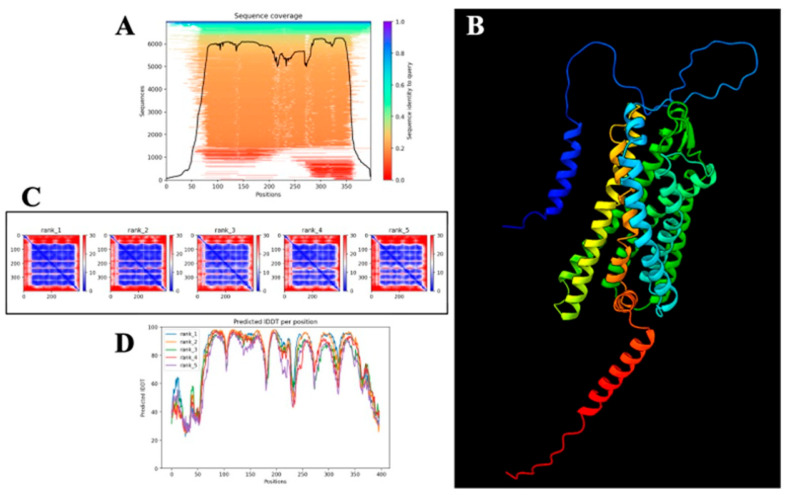
AlphaFold-based structural prediction of PAR-2 confirms canonical GPCR topology and supports downstream docking analysis. (**A**) Sequence coverage and per-residue confidence (pLDDT) plot from AlphaFold prediction showing high confidence scores (>90) across transmembrane domains, with lower confidence regions corresponding to extracellular and intracellular loops; (**B**) Three-dimensional ribbon model of human PAR-2 generated using AlphaFold, revealing the typical seven-transmembrane α-helical architecture characteristic of Class A GPCRs, with helices arranged to form a central ligand-accessible cavity; (**C**) Contact distance matrices for the top five ranked models, illustrating inter-residue spatial congruency and stability of the predicted transmembrane core; (**D**) Predicted IDDT score per residue across the five AlphaFold models, demonstrating consistent high-confidence predictions in helical regions and variability in terminal and loop regions.

Figure 16A shows the per-residue confidence scores (pLDDT) plotted alongside sequence coverage. The AlphaFold model demonstrated high-confidence predictions (pLDDT > 90) across the transmembrane helices, which constitute the core of the receptor’s functional architecture. The extracellular N-terminal domain and some loop regions exhibited lower pLDDT values, reflecting intrinsic flexibility and conformational heterogeneity commonly observed in GPCR extracellular domains.

The predicted 3D structure of PAR-2 is illustrated in Figure 16B. The model adopts a canonical Class A GPCR fold, comprising seven transmembrane α-helices organized in a right-handed bundle, three extracellular loops (ECL1–3), and three intracellular loops (ICL1–3). The helices form a central cavity—spanning helices III, V, VI, and VII—characteristic of ligand-binding domains in GPCRs. The arrangement and orientation of the transmembrane helices are structurally analogous to those reported for crystallized Class A GPCRs, underscoring the model’s fidelity. The cytoplasmic C-terminal tail, which typically exhibits conformational disorder, remained unresolved beyond a certain length, as expected from its unstructured nature.

To assess the robustness and internal consistency of the structural predictions, residue–residue distance contact maps were generated for the top five AlphaFold-ranked models (Figure 16C). These matrices display high concordance in inter-residue distances, particularly within the transmembrane core, indicating that the transmembrane bundle is reproducibly predicted across models.

Figure 16D illustrates the predicted IDDT (inter-residue distance difference test) scores per residue for all five models. The overlay of curves reveals a tightly clustered confidence profile, especially within the transmembrane segments, validating the reproducibility and reliability of the spatial predictions across multiple independently ranked models.

Taken together, the AlphaFold-generated model of PAR-2 provides a high-confidence structural framework consistent with a functional GPCR capable of ligand binding. The central cavity formed within the transmembrane helices presents a plausible and biophysically relevant site for small molecule interaction. This structural prediction was thus employed in subsequent molecular docking analyses to assess curcumin binding using the CB-Dock2 platform (refer below).

### 3.9. Molecular Docking Suggests Direct Interaction of Curcumin with PAR-2 at a Deep Transmembrane Pocket

Given the pronounced downregulation of PAR-2 expression by curcumin in both HT 29 and Caco-2 cell lines, coupled with reduced downstream signaling via ERK1/2 and reactivation of apoptotic cascades (*caspase-8* and -3), we sought to investigate whether curcumin may directly interact with PAR-2 at a structural level. To this end, molecular docking studies were conducted using the AlphaFold-predicted structure of human PAR-2 (refer Figure 16), followed by blind docking through the CB-Dock2 platform. This approach allowed identification of energetically favorable curcumin-binding pockets, enabling structure-based insights into its potential inhibitory mechanism.

Among five identified binding cavities (C1–C5), the site designated as C4 yielded the most favorable vina docking score of −6.9 kcal/mol, compared to −6.5, −6.1, −6.0, and −3.5 kcal/mol for the remaining sites, respectively. A more negative vina docking score reflects a stronger predicted binding affinity between the ligand and the receptor, indicating greater thermodynamic favourability of the interaction.

**Figure 17:**
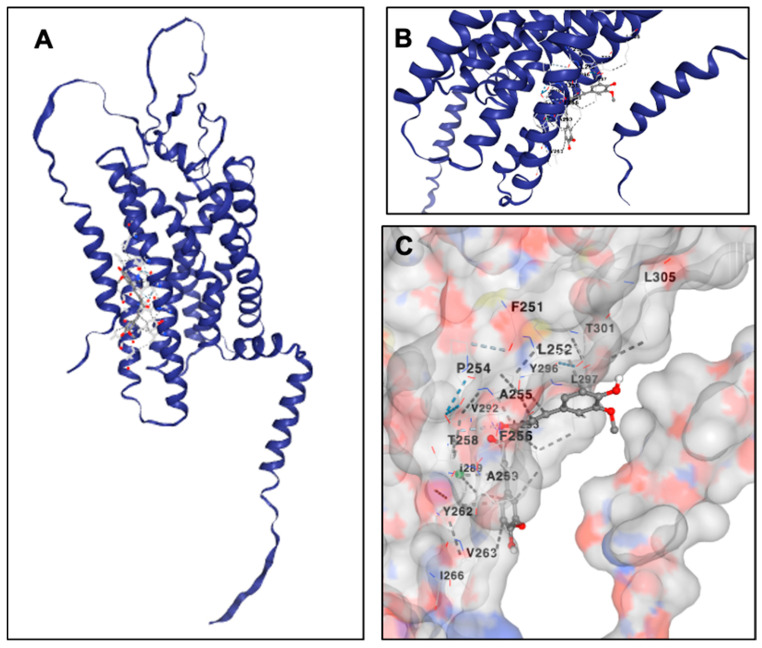
Molecular docking of curcumin with AlphaFold-modeled human PAR-2 structure using CB-Dock2 (**A**) Cartoon representation of the full-length PAR-2 receptor (predicted by AlphaFold2) showing curcumin bound within the C4 binding cavity, the site with the most favorable vina docking score (−6.9 kcal/mol); (**B**) Zoomed-in view of the transmembrane domain of PAR-2 depicting the docking pose of curcumin stabilized within the ligand-accessible pocket formed by helices TM3, TM5, and TM6; (**C**) Surface representation of the binding site highlighting specific interacting residues including F251, L252, A255, F256, T258, Y262, V263, and I266, suggesting a hydrophobic and hydrogen bonding–stabilized interaction network between curcumin and the PAR-2 binding groove.

C4 was characterized by a compact pocket (volume = 324 Å^3^) located within the transmembrane domain, centered at coordinates (13, 2, 11), and exhibited a hydrophobic microenvironment conducive to aromatic ligand binding. Based on the superior binding energy and spatial proximity to the functional core of PAR-2, this conformation was selected for detailed structural visualization and interaction profiling (Figure 17).

The 3D docking pose in Figure 17A demonstrates the localization of curcumin within the central transmembrane (TM) cleft of PAR-2. Figure 17B provides a lateral view, highlighting its insertion within helices TM3, TM5, and TM6. Notably, the ligand formed stable hydrophobic and van der Waals interactions with several conserved residues, including F251, L252, P254, A255, F256, Y262, and V263, all of which line the inner binding groove (Figure 17C). Additionally, putative hydrogen bonds and π–π stacking interactions with residues Y296 and T301 likely enhance binding stability, further anchoring curcumin in a functionally relevant site that may allosterically hinder signal transduction.

This docking model supports our experimental hypothesis that curcumin directly engages PAR-2 to suppress its signaling function. Given the spatial positioning within a region implicated in G-protein coupling and activation, the results raise the possibility of a steric hindrance or conformational locking mechanism by which curcumin exerts its anti-inflammatory and pro-apoptotic effects. These insights reinforce the notion that curcumin acts not merely as a transcriptional modulator of PAR-2 expression but may also function as a direct structural antagonist under inflammatory conditions relevant to colorectal cancer progression.

## 4. Discussion

In this study, we demonstrate that curcumin, a bioactive polyphenol, potently attenuates LPS-driven inflammatory signaling in CRC cells by downregulating PAR-2 and its downstream effectors, thereby reactivating apoptotic pathways. Curcumin treatment counteracted the LPS-induced upregulation of PAR-2 at both protein and mRNA levels in HT 29 and Caco-2 cells (Figure 2), without affecting the closely related PAR-1 (Figure 3). This selective PAR-2 inhibition translated into a suppression of mitogen-activated protein kinase (MAPK) signaling, as evidenced by reduced total ERK1/2 and phosphorylated ERK levels in both cell lines (Figure 4 and Figure 5). Consequently, curcumin blunted the production of the pro-inflammatory cytokine TNF-α (Figure 6) and relieved the PAR-2-mediated blockade of apoptosis. We observed restored expression of *caspase-8* (Figure 7), upregulation of the pro-apoptotic *Bax* (Figure 8) with concomitant downregulation of anti-apoptotic *Bcl 2* (Figure 9), and increased *caspase-3* expression (Figure 10). These molecular changes culminated in a marked induction of apoptosis, as confirmed by acridine orange/ethidium bromide staining (Figure 11), Annexin V/PI flow cytometry (Figure 12), and loss of cell viability in MTT assays (Figure 13). Notably, curcumin also prevented the LPS-triggered rise in intracellular Ca^2+^ (Figure 14), suggesting an additional mechanism by which curcumin may dampen inflammatory signaling. Finally, integrative in silico analyses indicate that curcumin may directly interact with PAR-2. In fact, an AlphaFold2-derived PAR-2 structure (Figure 16) revealed a credible ligand-binding pocket within the transmembrane (TM) domain, and molecular docking pinpointed a deep cavity where curcumin binds with high affinity (~−6.9 kcal/mol) (Figure 17). Below, we discuss each of these findings in the context of the current literature, focusing on the mechanistic sequence PAR-2 → ERK1/2 → pERK → TNF-α → *Caspase-8* → *Bax*/*Bcl 2* → *Caspase-3* → Apoptosis, and explore the translational implications and future directions of targeting the PAR-2 pathway in CRC.

Our results establish PAR-2 as a key target of curcumin’s action in inflamed CRC cells. Curcumin dose-dependently reversed the LPS-induced PAR-2 upregulation in both cell lines (Figure 2A–F). In contrast, the structurally homologous PAR-1 remained unchanged (Figure 3A–F), underscoring that curcumin’s effect is not due to a nonspecific shutdown of all PARs, but rather a selective modulation of the PAR-2 axis. This finding is novel and significant, as PAR-2 is increasingly recognized as an important pro-tumorigenic, pro-inflammatory receptor in CRC pathophysiology [147]. PAR-2 is often overexpressed in colorectal tumors and drives inflammatory cascades (e.g., ERK1/2 and PI3K/Akt pathways) that promote cancer cell proliferation, survival, and invasion [53]. By dampening PAR-2 expression, curcumin effectively short-circuits these oncogenic signals at their origin. Indeed, following PAR-2 suppression, we observed a marked reduction in downstream ERK1/2 signaling activity in curcumin-treated cells (Figure 4 and Figure 5). LPS alone robustly activated ERK1/2 (via TLR4-PAR-2 crosstalk) in our model, consistent with the known role of PAR-2 in amplifying MAPK signaling during inflammation [53,147]. Curcumin co-treatment significantly lowered total ERK1/2 protein levels and their phosphorylation (pERK), which aligns with curcumin’s well-documented inhibition of MAPK pathways in various cell types [148]. For example, curcumin has been reported to suppress ERK activation in immune cells and cancer cells as part of its broad anti-inflammatory, anti-proliferative effects [148,149]. Our findings mirror those in human mast cells where curcumin blocked PAR-2–mediated degranulation by inhibiting the downstream ERK pathway rather than by directly inactivating the protease agonist [149]. Thus, both in immune and epithelial contexts, curcumin appears to target the receptor→MAPK link as a means of controlling inflammation.

It is worth noting an intriguing nuance in the ERK signaling data. In HT 29 and Caco-2 cells, the ratio of phosphorylated ERK to total ERK was paradoxically elevated at the highest curcumin dose (Figure 4D and Figure 5D), despite an absolute reduction in both measures. One interpretation is that curcumin more strongly suppressed new ERK protein synthesis than it did ERK phosphorylation of the residual pool, resulting in a higher pERK/ERK ratio. This effect could arise from curcumin’s pleiotropic actions on transcriptional and post-translational regulation of ERK signaling components, such as its reported inhibition of transcription factors AP-1 and Egr-1, leading to reduced ERK1/2 transcription [98]. Concurrently, curcumin may alter the activity or expression of ERK-directed phosphatases like DUSP6, preserving phosphorylated ERK species within specific subcellular compartments (e.g., nuclear pools) even while total ERK declines [150]. This could reflect a compensatory feedback—for instance, reduced ERK levels might lessen the activation of ERK-responsive phosphatases like DUSP6 [151], causing the remaining ERK to retain phosphorylation. In line with this, there may be a plausible decrease in DUSP6 expression (an inducible ERK phosphatase) with curcumin treatment, reflected in the concomitant drop of ERK1/2 mRNA [151,152] (Figure 4 and Figure 5), suggesting curcumin curtailed the usual negative feedback loop. Additionally, curcumin’s induction of oxidative and endoplasmic reticulum (ER) stress could transiently activate upstream stress kinases, allowing residual ERK molecules to remain phosphorylated as part of a stress-adaptive response, a phenomenon observed in other models of curcumin-induced apoptosis [153]. Such complexity underscores the layered regulation of MAPK signaling. It is a possibility that curcumin’s partial inhibition of the pathway may unmask feedback dynamics that maintain a fraction of ERK in the active state. Nonetheless, the net effect clearly favored signaling suppression, as total pERK levels fell and downstream inflammatory outputs were reduced. This interpretation is further supported by the induction of apoptosis-related factors in the present investigation (e.g., *caspase-8* and *Bax*/*Bcl 2* modulation) downstream of ERK attenuation, indicating that the apparent ratio increase does not equate to functional pathway activation but rather to a redistribution of residual signaling pools under curcumin’s influence. Further studies could explore this phenomenon. This phenomenon can be investigated, through time-resolved phosphoproteomics [154], or mathematical modeling of the ERK pathway [155] to determine if curcumin induces any adaptive resistance in signaling networks. Overall, our data firmly support that curcumin attenuates the pro-inflammatory ERK cascade downstream of PAR-2. This is a critical mechanistic link, where by disconnecting PAR-2 from its MAPK effector, curcumin interrupts a major route by which PAR-2 drives gene expression of inflammatory and survival genes in CRC.

One important downstream consequence of PAR-2/MAPK signaling is the induction of cytokines like TNF-α. As expected, LPS stimulation sharply increased TNF-α secretion and mRNA in our cell models, and curcumin caused a dose-dependent blockade of this increase (Figure 6A–D). This finding is in agreement with the wealth of literature describing curcumin’s anti-NF-κB activity [156,157]. TNF-α expression in LPS-stimulated colon cells is largely NF-κB-dependent [158], and curcumin is a known inhibitor of NF-κB signaling [148]. By suppressing ERK and likely NF-κB activation (via TLR4/PAR-2), curcumin effectively prevented the transcriptional upregulation of TNF-α. Our observation parallels prior reports in macrophages and intestinal models where curcumin reduced LPS-induced TNF-α levels [159,160,161]. To further confirm the role of ERK in regulating TNF-α expression, we employed the selective ERK inhibitor SCH772984 (10 µM). Treatment with SCH772984 markedly reduced TNF-α secretion and mRNA levels in both HT 29 and Caco-2 cells (Figure 6E–H), supporting the conclusion that ERK signaling is a critical upstream regulator of TNF-α induction in this context. These findings strengthen our assertion that curcumin’s effect on TNF-α is mediated, at least in part, through ERK pathway suppression. This is significant because TNF-α not only propagates inflammation but also serves as a contextual signal for apoptosis. In cancer cells, TNF-α can have dual roles: it might induce apoptosis through extrinsic death receptors, but in an inflammatory milieu TNF-α often promotes survival by activating NF-κB and other pathways in tumor cells [162,163]. Here, curcumin’s simultaneous dampening of TNF-α and PAR-2/ERK suggests a coordinated shutdown of pro-survival inflammatory loops. The SCH772984 data also suggest that ERK inhibition reduces TNF-α-mediated survival signaling, thereby creating a cellular environment that is more permissive to *caspase-8* activation and the downstream *Bax*/*Bcl 2* apoptotic cascade. The net effect is that cells are no longer “primed” by inflammatory signals to resist apoptosis; instead, they become permissive to cell death pathways. Indeed, the positioning of the TNF-α results in our study was deliberate—having established that curcumin quelled upstream signals (PAR-2 and ERK), we showed TNF-α reduction as an intermediate outcome that “sets the stage” for downstream apoptotic effects. In essence, curcumin pulls multiple levers to tilt the balance from a pro-inflammatory, tumor-promoting state towards a pro-apoptotic state.

A central novel finding of our work is that curcumin reverses PAR-2-mediated apoptosis resistance, as evidenced by the robust upregulation of *caspase-8* (Figure 7). PAR-2 activation is known to confer resistance to apoptosis through sustained MEK1/2-ERK1/2 and PI3K-Akt signaling [57,139]. These pathways upregulate anti-apoptotic proteins and prevent the activation of caspases—for example, PAR-2 signaling can promote *BAD* phosphorylation and increase levels of survival proteins like MCL-1 and *Bcl2L12*, thereby impeding the proteolytic maturation of *caspase-8* [139]. Consistent with these reports, we observed that LPS (which indirectly activates PAR-2) kept *caspase-8* expression low (Figure 7A,D, lanes 2), indicating an apoptotic blockade at the initiator caspase level. Curcumin effectively lifted this blockade: *caspase-8* protein and mRNA were restored to, or even exceeded, baseline levels with curcumin co-treatment (Figure 7A–F). Although apoptosis is classically associated with depletion of the procaspase pool and accumulation of cleaved fragments, our concurrent observation of elevated full-length and cleaved caspase-8 suggests that curcumin transcriptionally upregulates *CASP8* while simultaneously promoting its activation, thereby replenishing substrate and ensuring sustained apoptotic signaling.Importantly, analysis of cleaved *caspase-8* (Figure 7G,H) revealed a marked increase in the active form of the enzyme with curcumin, confirming that curcumin not only restores *caspase-8* expression but also drives its proteolytic activation, a critical step in initiating the extrinsic apoptotic cascade. This suggests that curcumin negated the survival signals maintaining *caspase-8* suppression. Our data align with earlier findings that antagonizing PAR-2 or its downstream pathways can reinstate *caspase-8* activity and sensitize cells to apoptosis [57]. Also, PAR-2-driven ERK/Akt signaling prevents *caspase-8* activation [139]. By inhibiting these pathways, curcumin allows *caspase-8* to accumulate and presumably become activated in the presence of TNF-α or other death signals. The observed increase in cleaved *caspase-8* complements the subsequent induction of *Bax* and *caspase-3* (Figure 8 and Figure 10), indicating that curcumin coordinates the activation of both extrinsic and intrinsic apoptotic pathways in CRC cells. This pro-apoptotic shift induced by curcumin is particularly relevant in the context of cancer therapy. Many tumors, including CRC, upregulate PAR-2 and other inflammation-linked signals to evade apoptosis and resist chemotherapy [53]. Our results indicate that curcumin can counteract such mechanisms, thereby potentially resensitizing cancer cells to apoptotic triggers. Notably, curcumin’s effect on *caspase-8* was dose-dependent and reproducible in two distinct CRC lines, highlighting the robustness and generality of this mechanism.

Downstream of *caspase-8*, apoptosis signaling bifurcates into extrinsic and intrinsic pathways. *Caspase-8* can directly activate executioner caspases (extrinsic route) and can also cleave Bid to engage the mitochondrial (intrinsic) pathway [164]. We therefore examined *Bax* (pro-apoptotic) and *Bcl 2* (anti-apoptotic) as critical regulators of the intrinsic pathway. Curcumin dramatically upregulated *Bax* in LPS-treated cells (Figure 8) while downregulating *Bcl 2* (Figure 9), in both cases countering the LPS-induced pro-survival expression profile. In the presence of inflammation (LPS), cells tended to maintain low *Bax* and high *Bcl 2*, a hallmark of apoptosis resistance. Curcumin reversed this balance: the *Bax*/*Bcl 2* ratio was markedly increased in treated cells, favoring mitochondrial outer membrane permeabilization and apoptosis. These findings reinforce the notion that curcumin triggers apoptosis through multi-level regulation. Independent studies in other cancer models corroborate our observations. For example, in retinoblastoma cells, curcumin was shown to reduce *Bcl 2* and increase *Bax* and cleaved *caspase-3*/-9, driving the cells into apoptosis [148]. Likewise, in breast cancer and gastric cancer models, curcumin or its analogs increased the *Bax*/*Bcl 2* ratio to initiate cell death [165,166,167]. The consistency of this pattern across disparate cell types suggests that modulating *Bcl 2* family members is a common endpoint of curcumin’s pro-apoptotic action. Mechanistically, the drop in *Bcl 2* we see with curcumin could be a downstream effect of curcumin’s NF-κB inhibition, since *Bcl 2* is an NF-κB-responsive gene in many contexts [168]. It could also relate to the aforementioned *caspase-8* reactivation, as active *caspase-8* (via Bid cleavage) leads to mitochondrial engagement and can promote *Bax* activation. Interestingly, PAR-2 signaling has been linked to the maintenance of anti-apoptotic proteins (e.g., PAR-2 can induce MCL-1 and *Bcl2L12* [140]). Our data indicate that curcumin interrupts these signals, thereby lowering *Bcl 2* levels. The net effect is a cellular milieu that is decidedly pro-apoptotic. Also, high *Bax* expression induced by curcumin (Figure 8), drives mitochondrial cytochrome c release, concomitantly downregulation of *Bcl 2* by curcumin (Figure 9), fails to stop it, and caspase-9/3 can then be activated, as reflected in our measurements of *caspase-3*.

As the final executor of apoptosis, *caspase-3* was expected to be activated as a consequence of the upstream events. We indeed found that curcumin elevated *caspase-3* expression (Figure 10). Although we primarily measured the expression of *caspase-3* (full-length) rather than its cleaved fragments, the increase in *CASP3* mRNA, and protein with curcumin suggests that the cells were transcriptionally upregulating the executioner machinery, likely in response to the pro-apoptotic signaling cascade. In HT 29 cells, LPS alone did not significantly increase *caspase-3*, whereas curcumin did, implying that only curcumin-treated cells fully commit to the apoptotic program. In Caco-2, there was a slight increase in *caspase-3* protein with LPS, but it was not supported at the mRNA level, hinting at possible post-transcriptional stabilization of *caspase-3* under stress (a divergence we noted in results, Figure 10D–F). Regardless, in both cell lines the addition of curcumin caused a clear upsurge in *caspase-3*, consistent with the completion of the apoptotic cascade. This molecular evidence is powerfully reinforced by our functional apoptosis assays. Acridine orange/EtBr staining revealed that curcumin + LPS treatment led to widespread cell death (orange/red nuclei indicating late apoptosis/necrosis), whereas LPS alone left cells largely viable (green, Figure 11). Quantitation showed significant reductions in live cell density only when curcumin was present (Figure 11D,H). Similarly, Annexin V/PI flow cytometry demonstrated a major shift in cell populations into early and late apoptosis quadrants with curcumin treatment (Figure 12A–J). In both HT 29 and Caco-2, curcumin plus LPS caused ~3 to 4 fold increases in apoptotic cells compared to controls or LPS alone (Figure 12D,I), while LPS by itself caused minimal apoptosis—a non-significant change relative to untreated cells. These data confirm that LPS-induced inflammation is not intrinsically cytotoxic (consistent with our initial MTT and morphology observations, Figure 1B–E), but curcumin turns this inflammatory context into a lethal one for the cancer cells. The agreement among multiple orthogonal assays (microscopy, flow cytometry, biochemical markers) leaves little doubt that curcumin drives genuine programmed cell death rather than causing assay artifacts or generic toxicity. Notably, even the MTT viability assays showed that curcumin reduced cell viability in both the presence and absence of LPS (Figure 13A–D), whereas LPS alone had negligible impact on viability. This emphasizes that curcumin’s effect is dominant and that the inflammatory state (while promoting PAR-2 signaling and cytokine production) was primarily influencing *how* cells die rather than *if* they die. In absence of inflammation, curcumin still can kill CRC cells (through its well-known cytotoxic effects at high concentrations), but under inflammatory conditions relevant to the tumor microenvironment, curcumin’s pro-apoptotic efficacy is maintained or even enhanced due to the relief of inflammation-associated survival signals. Taken together, our findings paint a coherent picture: *curcumin dismantles the inflammation-induced survival network (PAR-2 → ERK → NF-κB/TNF-α → Bcl 2) that would normally shield CRC cells from apoptosis*, *thereby allowing both extrinsic and intrinsic apoptotic pathways to proceed to completion.* “*Although curcumin is known to modulate transcription factors like p53 and NF-κB in other models*, *our study did not directly assess these factors. The observed changes in Bcl 2/Bax and inflammatory markers are downstream indicators that align with p53/NF-κB influence*, *but we refrain from making any claims about p53 or NF-κB activation in the absence of direct assays*”.

We also discovered that curcumin abrogated LPS-induced intracellular Ca^2+^ elevation (Figure 14). This result provides an additional mechanistic insight. PAR-2, being Gq-coupled, and TNF-α can both trigger intracellular Ca^2+^ release as part of cell signaling [169,170]. In our model, LPS stimulation led to a pronounced Ca^2+^ flux (visualized by Fluo-4 fluorescence, Figure 14A–H), which is consistent with PAR-2 activation and inflammatory signaling. Elevated Ca^2+^ can activate numerous downstream pathways, including Ca^2+^-dependent kinases, calcineurin/NFAT, and even calpain-mediated apoptosis or necrosis, depending on context [171,172,173]. Curcumin’s ability to keep intracellular Ca^2+^ at baseline or below suggests that it interferes with this aspect of PAR-2/TLR4 signaling as well. One likely explanation is that by downregulating PAR-2 (and possibly affecting IP_3_ generation), curcumin prevents the G_q_—coupled calcium release from the endoplasmic reticulum that would normally occur upon PAR-2 stimulation [169]. Additionally, curcumin disrupts cellular Ca^2+^ homeostasis in cancer cells through other multifaceted mechanisms that reinforce its pro-apoptotic and anti-proliferative actions. It inhibits Orai1-mediated store-operated Ca^2+^ entry (SOCE), thereby impairing a critical influx pathway required for CRC cell growth and survival. Simultaneously, curcumin targets the mitochondrial Na^+^/Ca^2+^ exchanger (NCLX), promoting mitochondrial Ca^2+^ overload, respiratory dysfunction, and reactive oxygen species (ROS) generation—events that culminate in endoplasmic reticulum (ER) stress and apoptosis [174]. In parallel, curcumin enhances ER Ca^2+^ release by modulating inositol trisphosphate receptor (IP_3_R) activity, a process augmented by *Bcl 2* downregulation (*also observed in our study*, Figure 9) witnessed in non-small cell lung cancer models [175]. These concerted effects on Ca^2+^ influx, efflux, and ER-mitochondrial signaling establish curcumin as a dual modulator of Ca^2+^ dynamics. In malignant cells, this dyshomeostasis drives cytotoxicity, while in non-malignant contexts, it may offer cytoprotective benefits by attenuating pathological Ca^2+^ signaling. Together, these findings underscore curcumin’s capacity to reprogram Ca^2+^ signaling networks across intracellular compartments, coherently aligning with its anti-tumor mechanism of action. In our context, curcumin suppressing the LPS-induced Ca^2+^ rise might contribute to its overall anti-inflammatory effect (since Ca^2+^ is a secondary messenger for NF-κB activation and cytokine secretion [176]) and could also stabilize cellular physiology as apoptosis takes place. We noted that curcumin did not visibly perturb cell morphology aside from reducing fluorescence (Figure 14C,G), indicating it was not causing acute cytotoxic calcium shock, but rather normalizing calcium levels. Therefore, while the Ca^2+^ findings are ancillary, they reinforce the concept that curcumin globally dampens the “activated” state of the cell induced by LPS. This comprehensive suppression from calcium surges to kinase activation to cytokine releasecreates conditions conducive to apoptosis and detrimental to survival.

In our study, we observed slight differences *n* the response to curcumin between HT 29 and Caco-2. This differential response of HT 29 and Caco-2 cells to curcumin can be attributed to their distinct biological phenotypes and signaling landscapes. Caco-2 cells display markedly higher basal expression of PAR-2 compared to HT 29 cells, as reported by Darmoul et al. [64] and supported by our densitometric analysis (Figure 1A). This elevated PAR-2 abundance may enhance curcumin’s downregulatory effects on the PAR-2/ERK/TNF-α signaling axis. Furthermore, Caco-2 cells possess the ability to differentiate into enterocyte-like cells with brush-border morphology and tight junctions, closely resembling intestinal epithelial barrier function [177,178]. In contrast, HT 29 cells remain less differentiated and exhibit a more stem-like phenotype with enhanced proliferative capacity and resistance to apoptosis [179]. These intrinsic differences may influence the sensitivity of the mitochondrial and death receptor-mediated apoptotic pathways, particularly involving *caspase-3* and *caspase-8* activation, upon curcumin treatment. Additionally, the two cell lines display variations in Wnt/β-catenin signaling and calcium homeostasis, both of which intersect with PAR-2 pathways. Reports suggest that differentiated Caco-2 cells have a more stabilized β-catenin signaling profile and distinct MAPK/ERK activation patterns compared to HT 29 [180]. Such differences in intracellular signaling and NF-κB activity may underlie the subtle variations in cytokine secretion and apoptotic responses observed in our study. Together, these data highlight that intrinsic PAR-2 abundance, differentiation status, and signaling context significantly shape CRC cell line responsiveness to curcumin.

To complement these experimental findings, we employed structural modeling and molecular docking to determine whether curcumin might exert a direct antagonistic effect on PAR-2 at the receptor level. While our data clearly demonstrate transcriptional and translational downregulation of PAR-2 and its downstream effectors, the rapid attenuation of signaling events such as ERK phosphorylation and calcium flux within 24 h suggests an additional layer of acute receptor modulation. Structural prediction of PAR-2 using AlphaFold revealed a canonical GPCR fold with a well-defined transmembrane binding cleft, which was further interrogated by docking analyses with curcumin. The docking results indicate that curcumin may insert into a hydrophobic transmembrane pocket in a manner capable of stabilizing an inactive PAR-2 conformation. Such conformational locking would impede G-protein coupling and β-arrestin recruitment, thereby silencing downstream pathways, including ERK/MAPK and NF-κB signaling, which regulate transcription of TNF-α, *Bcl 2*, and other pro-survival factors. As illustrated in our integrative model (Figure 15), this structural interference could act synergistically with curcumin’s transcriptional suppression of PAR-2, amplifying its ability to disconnect the inflammatory-to-apoptotic signaling cascade. Thus, the in silico findings not only provide a plausible molecular explanation for the observed attenuation of PAR-2 signaling but also underscore a dual mechanism of action where curcumin functions both as a transcriptional regulator and as a potential structural antagonist of PAR-2.

To delve deeper into whether curcumin might directly bind and antagonize PAR-2, we turned to structural modeling and docking. Using AlphaFold2, we generated a high-confidence model of human PAR-2 (Figure 16), which displayed the canonical Class A GPCR topology: seven TM helices forming a central ligand-binding cavity, with flexible extracellular loops [49] (as evidenced by lower pLDDT confidence in those regions). The model’s TM core was predicted with high confidence and showed excellent agreement with known GPCR structures (e.g., closely overlapping helices in the distance contact maps across the top five models). This gave us a reliable scaffold to explore ligand binding. Blind docking of curcumin using the CB-Dock2 algorithm revealed five candidate binding pockets (C1–C5) on PAR-2, of which one pocket (denoted C4) was clearly the most energetically favorable. Curcumin docking in C4 yielded a vina score of approximately −6.9 kcal/mol, more negative (stronger binding) than the next-best pockets (scores −6.5, −6.1, −6.0, and −3.5 kcal/mol for pockets C1, C2, C3, C5, respectively) (Table 2). The C4 pocket is located deep in the transmembrane bundle, with an approximate volume of 324 Å^3^. Notably, this pocket is a hydrophobic cleft formed by helices III, V, VI (and likely VII)—aligning well with where many Class A GPCR antagonists bind allosterically or orthosterically. The docked pose (Figure 17A,B) showed curcumin nestled in this cleft, oriented roughly horizontal to the plane of the membrane. Curcumin’s aromatic rings engaged in hydrophobic contacts with multiple conserved residues lining the pocket, including F251, L252, P254, A255, and F256 on TM5 and Y262 and V263 on TM6. Additionally, the docking predicted potential hydrogen bonds or π–π stacking interactions between curcumin’s β-diketone or phenolic groups and residues Y296 and T301 (likely on TM7/ECL3). These interactions would together stabilize curcumin in a key region of the receptor. Intriguingly, the pocket identified is adjacent to the intracellular side of the receptor—i.e., near where the G-protein or β-arrestin would bind. If curcumin occupies this cavity, it could sterically hinder conformational changes or allosterically lock PAR-2 in an inactive state, preventing it from coupling to G-proteins. This structural insight dovetails nicely with our experimental data: it suggests that beyond downregulating PAR-2 gene expression, curcumin might acutely inhibit PAR-2 function by directly binding the receptor. In practical terms, curcumin could act as an allosteric antagonist of PAR-2 signaling, explaining why PAR-2 downstream effects (ERK phosphorylation, calcium release, NF-κB activation) were all inhibited even within 24 h of curcumin treatment—a timeframe in which direct receptor inhibition could play a role before full protein downregulation occurs.

It must be emphasized that this docking model, while compelling, is a computational prediction. Nonetheless, the notion of a small-molecule binding to PAR-2’s TM core is supported by emerging research. A notable example is GB88, a synthetic PAR-2 antagonist that has been shown to bind within the PAR-2 helical bundle and inhibit signaling. GB88 blocks PAR-2-mediated calcium release with an IC_50_ of ~2 μM and is effective in vitro and in vivo as an allosteric antagonist [181]. A recent cryo-EM structure of PAR-2 bound to an antagonist (PDB 9E7R) revealed that small molecules can occupy a cavity in the transmembrane region, stabilizing the inactive conformation of the receptor [144]. Although curcumin is structurally distinct from GB88, our docking-predicted binding site for curcumin overlaps with the general region targeted by known PAR-2 antagonists. Thus, our computational findings lend credence to a model where curcumin directly engages PAR-2 to suppress its activity. This dual mechanism, i.e., “direct receptor antagonism and transcriptional downregulation”, would make curcumin particularly effective at silencing PAR-2 signaling. It is interesting to speculate that curcumin’s polyphenolic structure, despite not being a drug-like optimized molecule, has just the right planar conformation and hydrophobic character to slip into GPCR pockets. Indeed, many natural polyphenols are promiscuous binders of proteins due to their hydrophobic rings [182], and in the case of PAR-2, this promiscuity might serendipitously translate into a specific inhibitory interaction. Future studies employing biophysical assays (e.g., those involving surface plasmon resonance [183], or isothermal titration calorimetry [184] with purified PAR-2) or mutagenesis of the putative binding residues could validate this interaction. We have planned such biophysical studies to directly investigate whether curcumin binds PAR-2 in vitro. Techniques such as SPR, isothermal titration calorimetry (ITC), and NMR spectroscopy are ideally suited for characterizing small molecule–GPCR interactions in native-like environments. For example, SPR has been successfully applied to detect ligand binding to GPCRs with preserved topology and membrane context [185], while ligand-observed NMR can reveal interactions with GPCRs even in complex systems [186]. These approaches will allow us to determine the affinity, kinetics, and potential allosteric nature of curcumin–PAR-2 binding without requiring receptor overexpression or perturbing its conformation. Validating curcumin’s binding via these methods would greatly strengthen the case for direct PAR-2 antagonism and could guide future drug design based on curcumin’s scaffold.

The ability of curcumin to downregulate PAR-2 and tip the balance towards apoptosis has important implications for CRC therapy. Chronic inflammation (such as in IBD) is a known risk factor for CRC progression, and PAR-2 is a critical link between inflammation and cancer in the colon. By curbing PAR-2, one can potentially break the inflammatory feedback loop that fosters tumor growth and also remove a survival advantage of cancer cells. This raises the prospect of using PAR-2 inhibitors, or agents like curcumin, as adjuvants to conventional chemotherapy. Most chemotherapeutic drugs (e.g., 5-fluorouracil, oxaliplatin) ultimately act by inducing sufficient DNA damage or stress to trigger cancer cell apoptosis. However, if cancer cells are under the influence of inflammatory pro-survival signals (like PAR-2 → ERK → NF-κB), they become more refractory to chemo-induced apoptosis. Our results show curcumin can disable these signals, suggesting that combining curcumin with cytotoxic drugs could yield a more effective tumor kill. There is already evidence to support this: in vitro and in vivo studies have demonstrated that curcumin enhances the efficacy of 5-FU against CRC cells. Du et al. [187] and Srimuangwong et al. [188] found that curcumin or its metabolite hexahydrocurcumin synergized with 5-FU to reduce colon tumor cell growth and increase apoptosis, both in culture and in mouse models. Curcumin also potentiated the pro-apoptotic, anti-metastatic effects of capecitabine (a 5-FU prodrug) in orthotopic models [189]. Furthermore, a recent phase I clinical trial reported that adding curcumin to the FOLFOX regimen (5-FU plus oxaliplatin and leucovorin) in metastatic CRC patients was safe and showed enhanced anti-proliferative effects ex vivo in patient-derived tumor explants [190,191]. These studies, together with our mechanistic data, indicate that curcumin can chemosensitize cancer cells—likely by lowering the threshold for apoptosis through pathways such as PAR-2 inhibition. It is tempting to draw parallels with other targeted therapies, just as blocking EGFR or PI3K can overcome resistance to apoptosis in certain tumors, blocking an inflammation-specific target like PAR-2 could make tumors more susceptible to immune or chemo-mediated killing. Indeed, beyond chemo, one could envision curcumin or PAR-2 antagonists improving responses to immunotherapy. PAR-2 in the tumor microenvironment influences cytokine profiles and immune cell recruitment [192], and therefore inhibiting PAR-2 might create a more favorable milieu for immune attack on the tumor. While our study did not directly address immune aspects (being an in vitro cell study), it underscores the value of targeting inflammatory GPCRs in cancer.

A noteworthy concern in drug development is that curcumin has been criticized as a “PAINS” (pan-assay interference compound) and an “IMPS” (invalid metabolic panacea) candidate [193,194], implying its multitudinous in vitro effects might be artifacts of assay interference (fluorescence, redox cycling, aggregation, etc.) rather than true target-specific actions [195]. Our findings help refute this concern by providing ‘multi-tiered validation’ of curcumin’s bioactivity on a specific pathway. First, the effects of curcumin we report are highly specific (e.g., selective downregulation of PAR-2 but not PAR-1; suppression of inducible targets like TNF-α, but no cytotoxicity to unstimulated cells at comparable doses). Such specificity is hard to reconcile with a non-specific PAINS mechanism, which would typically cause widespread assay signals (or false positives even in control conditions). Second, we used endpoint assays that are not easily fooled by common interference mechanisms. For instance, our qPCR measurements of gene expression are not susceptible to curcumin’s autofluorescence or chemical reactivity—they unequivocally show changes in mRNA levels for PAR-2, TNF, caspases, etc., in curcumin-treated cells. Similarly, Western blots demonstrate changes in protein levels based on immunodetection, which, aside from extremely high doses potentially causing off-target protein crosslinking, is a fairly interference-robust method. The consistency between mRNA and protein changes (e.g., curcumin decreased PAR-2 transcript and protein in tandem, Figure 2A,C) further supports genuine modulation of cellular pathways rather than an assay artifact. Third, the ultimate proof of pharmacologic effect is a functional outcome: we showed curcumin drives apoptosis as evidenced by morphological and flow cytometric criteria. The dramatic apoptotic phenotypes (nuclear condensation, Annexin V positivity) cannot be explained by simple assay interference and are in line with curcumin’s mechanistic effects upstream. Notably, we did control for curcumin’s natural fluorescence in flow cytometry—Annexin V-FITC and PI were chosen such that any fluorescence overlap from curcumin (which fluoresces in the blue/green range) would not be misinterpreted as a positive signal; appropriate compensation and gating were applied. Thus, the flow cytometry data reliably indicate phosphatidylserine externalization and membrane permeability changes due to apoptosis. All these pieces form a coherent story that validates curcumin’s activity in our system. Supporting this, numerous peer-reviewed studies over the last decade have documented specific molecular effects of curcumin in cancer cells—for example, curcumin consistently has been shown to downregulate *Bcl 2* and upregulate *Bax*, p53, and pro-apoptotic factors in various cancers, as we also see here. Such reproducibility across studies, cell types, and endpoints indicates that curcumin’s effects are real and biologically meaningful, even if its precise molecular targets can be multiple. The PAINS label serves as a caution to investigators to employ proper controls (which we did) and not overinterpret curcumin as a magic bullet. We acknowledge curcumin is not a highly selective drug—it may hit several targets and pathways (indeed our data suggest multi-target action, from TLR4/PAR-2/NF-κB to intrinsic apoptosis regulators). However, this multi-target nature can be an advantage in treating complex diseases like cancer, provided we understand the mechanism, which our work contributes to. In summary, our results argue that curcumin’s anti-inflammatory and pro-apoptotic actions in CRC are genuine and mechanism-based. This lays a foundation for taking curcumin (or its analogs) seriously in translational development, albeit with eyes open about its challenges.

## 5. Limitations and Future Directions

Our study adopts a reductionist in vitro approach, which, while powerful for mechanistic dissection, imposes several translational limitations. First, the use of 2D monolayer CRC lines (HT-29, Caco-2) cannot fully recapitulate the 3D tumor microenvironment, which in vivo involves stromal protease release, immune–tumor crosstalk, and paracrine PAR-2 activation [192]. Although our LPS model approximates inflammation (via NF-κB–mediated TNF-α upregulation), validation in 3D organoids or co-culture systems is warranted. Second, we relied solely on curcumin as a pharmacological inhibitor without complementary genetic manipulation. Curcumin’s polypharmacology—e.g., NF-κB inhibition, Nrf2 activation, ROS generation—means off-target effects cannot be excluded. While PAR-1 sparing and docking analyses support specificity, PAR-2 knockout/knockdown or use of selective antagonists (e.g., GB88) would strengthen causal attribution. Rescue experiments (e.g., PAR-2 overexpression or constitutive MEK activation) could further validate the pathway. Third, in vivo confirmation is absent. Curcumin’s bioavailability is limited, with <1% absorption and plasma levels rarely exceeding nanomolar concentrations, compared with the 50–100 µM used in vitro [193]. Although colonic tissue concentrations can reach higher micromolar levels, efficacy remains speculative. Animal models (e.g., AOM/DSS colitis-associated CRC) or xenografts with PAR-2 silencing are needed to assess translational relevance, alongside advanced formulations such as nanoparticle-encapsulated curcumin [196,197]. Fourth, assay-specific limitations exist: Western blots quantified total but not active protein (e.g., cleaved caspase-3, PARP), and cytokine analysis was restricted to TNF-α, omitting IL-6/IL-8. Finally, our docking analysis is predictive: although AlphaFold2-derived PAR-2 structures suggest curcumin binding (~−6.9 kcal/mol), docking assumes rigidity and modest affinity, necessitating validation via ITC, SPR, NMR, or mutagenesis.

Despite these constraints, the work highlights several promising avenues. Validation in CRC organoids and in vivo models could establish whether curcumin downregulates PAR-2 and induces apoptosis under physiologic conditions. Translationally, combining curcumin with chemotherapy (e.g., 5-FU, oxaliplatin) or immunotherapy could overcome inflammation-associated chemoresistance [190,191]. PAR-2 profiling may enable patient stratification, while nanoformulations could address pharmacokinetic barriers. Drug development efforts targeting PAR-2 are particularly compelling: selective antagonists such as GB88 provide proof-of-concept, and our docking results suggest a pharmacophore that could be refined via AI-driven medicinal chemistry [198,199]. This strategy could yield analogs with greater potency, specificity, and bioavailability, while exploiting curcumin’s polypharmacology. Systems-level approaches, including quantitative proteomics, single-cell transcriptomics, and ODE-based modeling, could map curcumin’s global effects, predict resistance mechanisms, and identify biomarkers of response [200,201]. Dynamic proteomic modeling, coupled with live-cell imaging of caspases, calcium flux, and mitochondrial potential, may further clarify thresholds and temporal dynamics of apoptosis induction.

In summary, while limited by in vitro design, absence of genetic manipulation, and pharmacokinetic hurdles, this work establishes PAR-2 as a credible therapeutic target in inflammation-driven CRC and positions curcumin as a scaffold for next-generation PAR-2–directed agents.

## 6. Conclusions

Our study delineates a mechanistic framework through which curcumin exerts anti-cancer effects in inflammation-driven CRC. By selectively downregulating PAR-2, while sparing PAR-1, curcumin disrupts the LPS–PAR-2 axis linking MAPK (ERK1/2) and NF-κB signaling, thereby reducing TNF-α expression, relieving the apoptotic blockade, and activating both extrinsic (caspase-8, cleaved caspase-8) and intrinsic (Bax/Bcl-2, caspase-3) pathways (Figure 2, Figure 3, Figure 4, Figure 5, Figure 6, Figure 7, Figure 8, Figure 9, Figure 10, Figure 11 and Figure 12). Restoration of calcium homeostasis (Figure 14) further underscores curcumin’s ability to redirect signaling toward apoptosis.

A notable strength is the integration of in vitro data with in silico structural modeling. AlphaFold2-derived PAR-2 models identified a hydrophobic pocket in which curcumin was predicted to bind with favorable affinity (~−6.9 kcal/mol) (Figure 16 and Figure 17). These docking results suggest that curcumin’s activity may extend beyond transcriptional downregulation to include allosteric GPCR modulation, potentially silencing PAR-2 signaling at the receptor level. However, this remains a predictive hypothesis requiring experimental validation (e.g., mutagenesis, ITC, SPR, NMR). Thus, curcumin may act via a dual mechanism of transcriptional repression and receptor antagonism, positioning it as a nutraceutical with GPCR-modulating potential.

Figure 15 summarizes this integrated mechanism, highlighting disruption of the PAR-2/ERK/NF-κB axis and convergence on apoptotic signaling nodes. Translationally, these findings underscore the therapeutic value of targeting PAR-2 to overcome inflammation-associated chemoresistance. While clinical use of curcumin is limited by poor bioavailability and PAINS concerns, our results support the rationale for developing curcumin analogs or PAR-2–specific small molecules as adjuncts in CRC therapy. Nevertheless, conclusions must be tempered by the in vitro context, and future research should focus on in vivo validation, PAR-2 genetic silencing, and AI-guided optimization of curcumin-inspired antagonists.

## Figures and Tables

**Table 1:** Oligonucleotide primers used for Real-Time PCR.

Genes for (Proteins)	Primer Type	Sequence	Accession	E-Value	Bit Score
*Bax*	Forward (5′-3′) Reverse (5′-3′)	TCAGGATGCGTCCACCAAGAAG TGTGTCCACGGCGGCAATCATC	NM_004324	0.0	1412
*Bcl 2*	Forward (5′-3′) Reverse (5′-3′)	ATCGCCCTGTGGATGACTGAGT GCCAGGAGAAATCAAACAGAGGC	BC027258	0.0	2704
*CASP3*	Forward (5′-3′) Reverse (5′-3′)	GGAAGCGAATCAATGGACTCTGG GCATCGACATCTGTACCAGACC	NM_004346	0.0	2645
*CASP8*	Forward (5′-3′) Reverse (5′-3′)	AGAAGAGGGTCATCCTGGGAGA TCAGGACTTCCTTCAAGGCTGC	NM_001080125	0.0	2929
*ERK 1*	Forward (5′-3′) Reverse (5′-3′)	TGGCAAGCACTACCTGGATCAG GCAGAGACTGTAGGTAGTTTCGG	NM_002746	0.0	1787
*ERK 2*	Forward (5′-3′) Reverse (5′-3′)	ACACCAACCTCTCGTACATCGG TGGCAGTAGGTCTGGTGCTCAA	NM_002745	0.0	5881
*GAPDH*	Forward (5′-3′) Reverse (5′-3′)	GTCTCCTCTGACTTCAACAGCG ACCACCCTGTTGCTGTAGCCAA	NM_002046	0.0	2374
*PAR-1*	Forward (5′-3′) Reverse (5′-3′)	GCTGTCCTACTGCTTGGAAGAC CTGCATCAGCACATACTCCTCC	NM_022002	0.0	2745
*PAR-2*	Forward (5′-3′) Reverse (5′-3′)	CTCCTCTCTGTCATCTGGTTCC TGCACACTGAGGCAGGTCATGA	NM_005242	0.0	2861
*pERK*	Forward (5′-3′) Reverse (5′-3′)	GTCCCAAGGCTTTGGAATCTGTC CCTACCAAGACAGGAGTTCTGG	NM_004836	0.0	4536
*TNF-α*	Forward (5′-3′) Reverse (5′-3′)	CTCTTCTGCCTGCTGCACTTTG ATGGGCTACAGGCTTGTCACTC	NM_000594	0.0	2449

Note: *PAR-2*: Protease Activated Receptor-2; *GAPDH*: Glyceraldehyde-3-phosphate dehydrogenase; *PAR-1*: Protease Activated Receptor-1; *pERK*: Phosphorylated Extracellular signal-regulated kinase; *ERK 1*: Extracellular signal-regulated kinase 1; *ERK 2*: Extracellular signal-regulated kinase 2; *CASP3* and *CASP8 *genes (encoding *caspase-3* and *caspase-8*, respectively); *Bax*: *Bcl 2*-associated X protein; *Bcl 2*: B-cell lymphoma 2.

**Table 2:** Docking Parameters and Scores for Curcumin Binding to Predicted PAR-2 Pockets Using CB-Dock2.

Pocket ID	Vina Score (Kcal/Mol)	Cavity Volume (Å^3^)	Center Coordinates (x, y, z)	Docking Box Size (x, y, z)
C4	−6.9	324	(13, 2, 11)	(26, 26, 26)
C1	−6.5	698	(−14, 10, 19)	(26, 26, 26)
C3	−6.1	353	(−6, 16, 1)	(26, 26, 26)
C2	−6.0	580	(−25, −6, 12)	(26, 26, 26)
C5	−3.5	283	(9, −8, −7)	(26, 26, 26)
Pocket ID	Vina Score (kcal/mol)	Cavity Volume (Å^3^)	Center Coordinates (x, y, z)	Docking Box Size (x, y, z)
C4	−6.9	324	(13, 2, 11)	(26, 26, 26)
C1	−6.5	698	(−14, 10, 19)	(26, 26, 26)
C3	−6.1	353	(−6, 16, 1)	(26, 26, 26)
C2	−6.0	580	(−25, −6, 12)	(26, 26, 26)
C5	−3.5	283	(9, −8, −7)	(26, 26, 26)
Pocket ID	Vina Score (kcal/mol)	Cavity Volume (Å^3^)	Center Coordinates (x, y, z)	Docking Box Size (x, y, z)
C4	−6.9	324	(13, 2, 11)	(26, 26, 26)
C1	−6.5	698	(−14, 10, 19)	(26, 26, 26)
C3	−6.1	353	(−6, 16, 1)	(26, 26, 26)
C2	−6.0	580	(−25, −6, 12)	(26, 26, 26)
C5	−3.5	283	(9, −8, −7)	(26, 26, 26)

Note: The docking box size refers to the dimensions of the 3D search grid used by AutoDock Vina for ligand exploration. All values are given in Ångströms (Å), where 1 Å = 10^−10^ m.

## Data Availability

The datasets generated and/or analyzed during the current study are not publicly available but are available from the corresponding author (YB) on reasonable request.

## Abbreviations

Akt	Protein kinase B
AO/EtBr	Acridine orange/ethidium bromide
AP-1	Activator protein 1
*APC*	Adenomatous polyposis coli
*Bax*	*Bcl 2*-associated X protein
*Bcl 2*	B-cell lymphoma 2
BIRC5	Baculoviral IAP repeat-containing protein 5 (Survivin)
*BRAF*	v-Raf murine sarcoma viral oncogene homolog B
CAC	Colitis-associated cancer
CB-Dock2	Cavity-detection guided protein–ligand docking tool
CIMP	CpG island methylator phenotype
CIN	Chromosomal instability
COX-1	Cyclooxygenase-1
COX-2	Cyclooxygenase-2
CRC	Colorectal cancer
CSCs	Cancer stem cells
DUSP6	Dual Specificity Phosphatase 6.
Egr-1	Early Growth Response 1.
ER	Endoplasmic reticulum
ERK	Extracellular signal-regulated kinase
*F2R*	Coagulation factor II (thrombin) receptor (gene encoding PAR-1)
*F2RL1*	Coagulation factor II (thrombin) receptor-like 1 (gene encoding PAR-2)
*F2RL2*	Coagulation factor II (thrombin) receptor-like 2 (gene encoding PAR-3)
*F2RL3*	Coagulation factor II (thrombin) receptor-like 3 (gene encoding PAR-4)
GPCR	G-protein coupled receptor
IBD	Inflammatory bowel disease
IKK	IκB kinase
IL-1β	Interleukin-1 beta
IL-6	Interleukin-6
JAK	Janus kinase
JNK	c-Jun N-terminal kinase
KRAS	Kirsten rat sarcoma viral oncogene homolog
LPS	Lipopolysaccharide
MAPK	Mitogen-activated protein kinase
*MLH1*	MutL homolog 1
MSI	Microsatellite instability
MTT	3-(4,5-dimethylthiazol-2-yl)-2,5-diphenyltetrazolium bromide
NF-κB	Nuclear factor-kappa B
ns	Not significant
p-ERK	Phosphorylated extracellular signal-regulated kinase
PAR-1	Protease-activated receptor-1
PAR-2	Protease-activated receptor-2
PARP	Poly (ADP-ribose) polymerase
PI	Propidium iodide
PI3K	Phosphoinositide 3-kinase
ROS	Reactive oxygen species
STAT3	Signal transducer and activator of transcription 3
TME	Tumor microenvironment
TNF-α	Tumor necrosis factor-alpha
*TP53*	Tumor protein p53
UC	Ulcerative colitis
VEGF	Vascular endothelial growth factor
